# Smarcd3 is an epigenetic modulator of the metabolic landscape in pancreatic ductal adenocarcinoma

**DOI:** 10.1038/s41467-023-35796-7

**Published:** 2023-01-18

**Authors:** L. Paige Ferguson, Jovylyn Gatchalian, Matthew L. McDermott, Mari Nakamura, Kendall Chambers, Nirakar Rajbhandari, Nikki K. Lytle, Sara Brin Rosenthal, Michael Hamilton, Sonia Albini, Martin Wartenberg, Inti Zlobec, José A. Galván, Eva Karamitopoulou, Vera Vavinskaya, Alexis Wascher, Andrew M. Lowy, Christian M. Schürch, Pier Lorenzo Puri, Benoit G. Bruneau, Diana C. Hargreaves, Tannishtha Reya

**Affiliations:** 1 Department of Pharmacology, University of California San Diego School of Medicine, La Jolla, CA USA; 2 Sanford Consortium for Regenerative Medicine, La Jolla, CA USA; 3 Salk Institute for Biological Studies, La Jolla, CA USA; 4 Center for Computational Biology and Bioinformatics, University of California San Diego School of Medicine, La Jolla, CA USA; 5 Genethon, 91000 EVRY, France; 6 Université Paris-Saclay, Univ Evry, Inserm, Genethon, Integrare research unit UMR_S951, 91000 Evry, France; 7 Institute of Pathology, University of Bern, Murtenstrasse 31, 3008 Bern, Switzerland; 8 Department of Pathology, University of California San Diego School of Medicine, La Jolla, CA USA; 9 Moores Cancer Center, University of California San Diego School of Medicine, La Jolla, CA USA; 10 Department of Surgery, Division of Surgical Oncology, University of California San Diego School of Medicine, La Jolla, CA USA; 11 Department of Pathology and Neuropathology, University Hospital and Comprehensive Cancer Center Tübingen, Tübingen, Germany; 12 Development, Aging and Regeneration Program, Sanford Burnham Prebys Medical Discovery Institute, La Jolla, CA USA; 13 Gladstone Institutes, Roddenberry Center for Stem Cell Biology and Medicine, San Francisco, CA USA; 14 Cardiovascular Research Institute, University of California San Francisco, San Francisco, CA USA; 15 Department of Pediatrics, University of California San Francisco, San Francisco, CA USA; 16 Department of Medicine, University of California San Diego School of Medicine, La Jolla, CA USA; 17 Present Address: Department of Physiology and Cellular Biophysics, Herbert Irving Comprehensive Cancer Center, Columbia University Medical Center, New York, NY 10032 USA

**Keywords:** Cancer epigenetics, Cancer metabolism, Cancer models, Cancer stem cells, Pancreatic cancer

## Abstract

Pancreatic cancer is characterized by extensive resistance to conventional therapies, making clinical management a challenge. Here we map the epigenetic dependencies of cancer stem cells, cells that preferentially evade therapy and drive progression, and identify SWI/SNF complex member SMARCD3 as a regulator of pancreatic cancer cells. Although SWI/SNF subunits often act as tumor suppressors, we show that SMARCD3 is amplified in cancer, enriched in pancreatic cancer stem cells and upregulated in the human disease. Diverse genetic mouse models of pancreatic cancer and stage-specific *Smarcd3* deletion reveal that *Smarcd3* loss preferentially impacts established tumors, improving survival especially in context of chemotherapy. Mechanistically, SMARCD3 acts with FOXA1 to control lipid and fatty acid metabolism, programs associated with therapy resistance and poor prognosis in cancer. These data identify SMARCD3 as an epigenetic modulator responsible for establishing the metabolic landscape in aggressive pancreatic cancer cells and a potential target for new therapies.

## Abstract (web summary)

Clinical management of pancreatic cancer remains challenging. Here, the authors suggest SMARCD3 as a potential epigenetic dependency establishing the metabolic landscape in aggressive pancreatic cancer cells and as a potential therapeutic target in pancreatic cancer.

## Introduction

Pancreatic ductal adenocarcinoma (pancreatic cancer, PDAC) is a highly lethal disease with poor clinical outcomes. Currently, the third leading cause of cancer-related deaths, pancreatic cancer has a 5-year survival rate of only 12% and is predicted to become the second leading cause in the United States by 2030^1,2^. Mortality is usually driven by characteristically late diagnosis, early metastasis, and resistance to conventional and targeted therapies^3^. Thus, understanding the molecular programs that underpin the growth of therapy-resistant cells remains a crucial priority for developing new strategies for pancreatic cancer treatment. Previous work has shown that therapy resistance is driven by differential responses to conventional agents fueled by the heterogeneity of tumor cells^4^; in particular, subpopulations that harbor stem cell characteristics are highly enriched for therapy resistance^5–9^. As in development, the undifferentiated state of these cells is driven in large part by epigenomic shifts rather than genomic changes^10^. But how these epigenetic changes are regulated, and how these regulatory programs shift as cancer cells become established during disease progression remains relatively unexplored. Given the reliance of these aggressive cells on epigenetic regulation, identifying chromatin-level drivers and the mechanisms by which they support the stem cell state in cancer is key to better understanding therapy resistance.

*Smarcd3* encodes the Baf60c subunit of SWI/SNF, a nucleosome remodeling complex that coordinates state-specific enhancers and is required for stem cell function in development^11^. This modular complex has many variable compositions, enabling the execution of cell state-specific programs by unique SWI/SNF assemblies^12^. Although a limited number of studies have identified cancer stem cell functions for SWI/SNF in vivo^13–16^, we are only beginning to understand how SWI/SNF subunits support stem cell fate and the mechanisms by which these chromatin remodelers control core functional programs in cancer. Further, as emerging research has revealed the highly context-specific roles of SWI/SNF subunits in cancer, determining how SWI/SNF dependencies vary across tissue and disease stages may enable the appropriate design of epigenetic therapies. As technology for targeting these proteins advances, identifying SWI/SNF subunits with stem-specific functions in cancer could have far-reaching impacts on cancer therapy^17,18^.

Here, we show that *Smarcd3* is upregulated in the stem cell fraction of mouse pancreatic tumors, and is further amplified and enriched in human pancreatic tumors^19^. Functionally, we use diverse stage-specific conditional genetic models to show that *Smarcd3* deletion specifically impacts established tumor growth, synergizing with chemotherapy to improve survival in tumors post-establishment. Consistent with this, SMARCD3 is required for the propagation of patient-derived tumors in vitro and in vivo. Mechanistically, comprehensive ChIP-seq and RNA-seq analysis shows that *Smarcd3* knockdown drives global losses in SWI/SNF binding and histone acetylation at active enhancers co-bound by FOXA1, downregulating a network of genes implicated in lipid homeostasis. Loss of *Smarcd3* blunts fatty acid metabolism in vivo, positioning SMARCD3 as an epigenetic regulator of fatty acid metabolism, a program which has been associated with stem cell signaling, therapy resistance, and poor prognosis in cancer^20^. Collectively these data identify SMARCD3 as a SWI/SNF subunit that is required for the growth of aggressive cancer stem cells and that exerts its influence by regulating the metabolic landscape in pancreatic cancer.

## Results

### SMARCD3 is a potential functional epigenetic dependency in pancreatic cancer

**Fig. 1:**
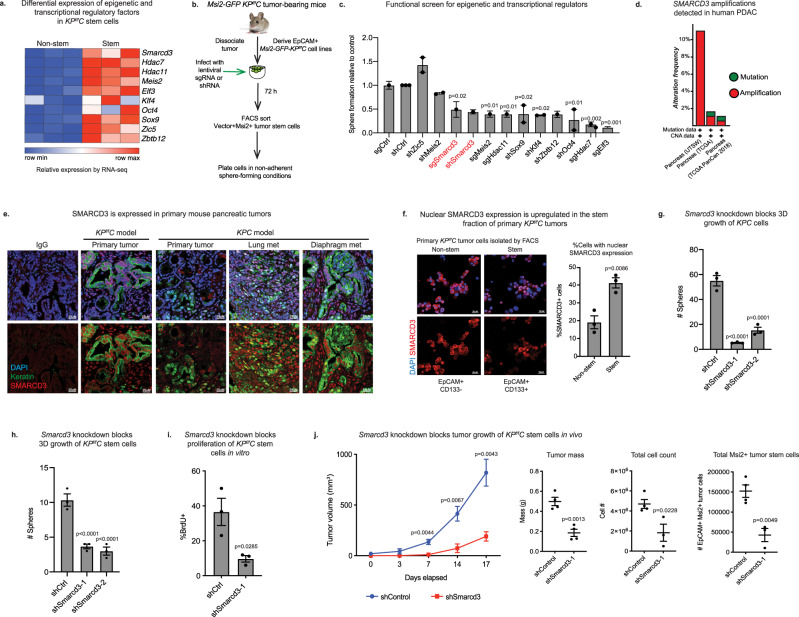
SMARCD3 is a potential functional epigenetic dependency in pancreatic cancer. **a** Relative expression of stem cell-enriched regulatory factors in primary stem (*Msi2-GFP*+) versus non-stem (*Msi2-GFP-*) EpCAM+ tumor cells by RNA-seq^8^. **b** Targeted 3D functional screen for dependencies of *Msi2-GFP KP*^*f/f*^*C* cells. **c** Functional screen identifies SMARCD3 as a dependency for PDAC stem cell growth. Relative sphere formation of MSI2+ *KP*^*f/f*^*C* cells normalized to control (*n* = 1 biological replicate at *n* = 3 for sgRNA and *n* = 3 biological replicates at *n* = 4 for shRNA; ANOVA with multiple comparisons, mean ± SEM). **d** Genetic amplifications in *SMARCD3* locus in clinical cases of pancreatic cancer (cBioPortal, see Supplementary Fig. 1a). **e** SMARCD3 expression in *KP*^*f/f*^*C* and *KPC* tumor cells. Representative images showing SMARCD3 (red) in epithelial tumor cells (pan-keratin+, green); nuclei (DAPI, blue), representative images (*n* = 3 mice, scale bar = 25 μm). **f** Elevated nuclear SMARCD3 in CD133+ stem cell fraction of primary *KP*^*f/f*^*C* tumors. DAPI (blue), SMARCD3 (red); tumor cells with nuclear SMARCD3 staining were counted. Representative from *n* = 3 frames, *n* = 2 biological replicates, mean ± SEM; scale bar = 25 μm (see Supplementary Fig. 1d). **g** *Smarcd3* knockdown blocks 3D growth of CD133 + *KPC* cells in vitro; *n* = 3, representative of *n* = 3 biological replicates, ANOVA with multiple comparisons, mean ± SEM. **h** *Smarcd3* knockdown blocks 3D sphere formation of CD133 + *KP*^*f/f*^*C* cells in vitro; *n* = 3, representative of *n* = 10 biological replicates, ANOVA with multiple comparisons, mean ± SEM. **i** *Smarcd3* knockdown blocks proliferation (BrdU incorporation) of CD133 + *KP*^*f/f*^*C* cells in vitro (*n* = 2 biological replicates, *n* = 3, mean ± SEM). **j** *Smarcd3* knockdown blocks growth of MSI2+ *KP*^*f/f*^*C* cells in vivo, reducing flank transplant tumor growth rate (shControl slope = 43.8 mm^3^/day; shSmarcd3 slope = 10.08 mm^3^/day, linear regression, *p* = <0.0001); mass, cell count, and number of MSI2+ tumor stem cells at endpoint (*n* = 4 for 3 biological replicates, ANOVA with multiple comparisons, mean ± SEM; see Supplementary Fig. 1i, j). Source data for all experiments are provided as a Source Data file.

To define epigenetic and transcriptional regulatory programs required for PDAC stem cell function, we used an RNA-sequencing (RNA-seq) dataset^8^ to identify factors significantly enriched in the therapy-resistant MSI2+ stem cell fraction^7^ of primary tumors from the *Kras*^*G12D/+*^*; p53* ^*f/f*^*; Ptf1a*-*Cre* (*KP* ^*f/f*^*C*) model of pancreatic cancer (Fig. 1a)^21–24^. To assess their impact, we conducted a targeted functional screen using primary cancer stem cells derived from *Msi2-GFP* reporter *KP* ^*f/f*^*C* tumors (Fig. 1b)^7^, where cells were transduced with lentiviral shRNA or sgRNA, and growth was analyzed in sphere-forming conditions^25^. Master transcription factors and histone deacetylases such as KLF4^26^, OCT4^27^, SOX9^28^, HDAC11^29^, and HDAC7^30^ were required for the growth of PDAC stem cells, serving as controls (Fig. 1c). Among genes not previously linked to pancreatic cancer, knockdown or deletion of *Smarcd3*, an SWI/SNF family member, reduced sphere formation of *KP* ^*f/f*^*C* stem cells by 50% (Fig. 1c). SMARCD3 was particularly interesting because it was the only significantly stem-enriched chromatin remodeling factor (FC > 2,FDR < 0.25), and because, unlike many other SWI/SNF subunits that are targeted for loss-of-function alterations^31^, *SMARCD3* was amplified in cancer (Fig. 1d, Supplementary Fig. 1a; cBioPortal^32,33^).

Consistent with a potential role in cancer, SMARCD3 was highly expressed in end-stage primary tumors from *KP* ^*f/f*^*C* mice, an aggressive model of pancreatic cancer driven by *p53* deletion^24^ (Fig. 1e). SMARCD3 was also expressed in both primary and metastatic lesions from the *Kras*^*G12D/+*^*; p53*^*R172H/+*^*; Ptf1a-Cre* (*KPC*) model, which recapitulates the metastatic behavior of the human disease^34^ (Fig. 1e). Further, although the core SWI/SNF subunit SMARCA4 was expressed in almost all primary stem and non-stem tumor cells (Supplementary Fig. 1b), nuclear SMARCD3 expression was upregulated within freshly isolated primary *KP* ^*f/f*^*C* CD133+ tumor cells, consistent with a role in the stem cell compartment (Fig. 1f, Supplementary Fig. 1c, d); CD133 is a cell surface marker commonly used to assess or isolate tumor stem cells by FACS^9^. Though some cytoplasmic SMARCD3 staining is apparent in *KP* ^*f/f*^*C* cells by cytospin (Fig. 1f), we focused solely on its expression in the nucleus, its primary site of action. Knockdown of *Smarcd3* mediated by two independent shRNAs reduced 3D growth of *KPC* and *KP* ^*f/f*^*C* cells by over 50% (Fig. 1g, h, Supplementary Fig. 1e–g), inhibiting proliferation and increasing cell death in vitro (Fig. 1i, Supplementary Fig. 1h). Further, shRNA-mediated knockdown of *Smarcd3* in MSI2+ *KP* ^*f/f*^*C* cells almost completely blocked flank tumor growth in NOD-SCID mice in vivo, reducing growth rate by over 4-fold, and total tumor cell and MSI2+ tumor stem cell counts by 2.5 and 3.5-fold (Fig. 1j, Supplementary Fig. 1i, j). As a corollary, we found that overexpression of *SMARCD3* in *KP* ^*f/f*^*C* cells increased their 3D growth by 2-fold and sustained the CD133+ fraction in vitro (Supplementary Fig. 1k–n), supporting an oncogenic function aligned with amplifications in the *SMARCD3* locus in PDAC^19^. These data collectively indicate that *Smarcd3* may represent a core dependency program for pancreatic cancer cells in transplant-based models.

### Genetic deletion of Smarcd3 impairs tumor growth in mouse models of pancreatic cancer

To better understand how *Smarcd3* contributes to the establishment and sustained propagation of cancer cells through the course of tumor progression in vivo, we used a diverse set of autochthonous genetic models to delete *Smarcd3* in a temporally restricted manner. To test how *Smarcd3* contributes to early pancreas cancer establishment, we crossed a conditional *Smarcd3*^*f/f*^ line^35^ to the *Kras*^*LSL/+*^; *Ptf1a-Cre* (*KC*) model, where embryonic activation of KRAS in pancreatic precursors drives the formation of benign PanIN lesions^23^, as well as the *Kras*^*LSL/+*^; *Ptf1a-Cre*^*ER*^ and *Kras*^*LSL/+*^; *Sox9-Cre*^*ER*^ ^36^ models, where benign lesions are initiated in adult acinar or ductal cells respectively. While embryonic *Smarcd3* deletion concomitant with RAS activation slightly promoted the emergence of benign cystic lesions arising from pancreatic progenitors (Supplementary Fig. 2a), *Smarcd3* deletion in adulthood with RAS activation did not significantly impact the development of early PanIN lesions arising from pancreatic cells of either lineage, indicating that *Smarcd3* does not play a key role in early tumor initiation (Supplementary Fig. 2a–c).

**Fig. 2:**
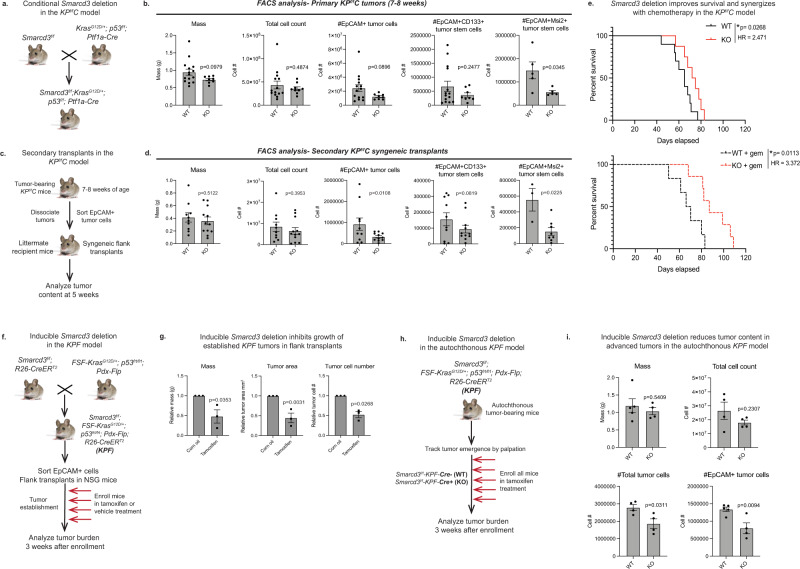
Genetic deletion of Smarcd3 impairs tumor growth in mouse models of pancreatic cancer. **a** Pancreas-specific deletion of *Smarcd3* concomitant with *Kras* mutation and *p53* deletion. **b** *Smarcd3* deletion in primary *KP*^*f/f*^*C* tumors. Analysis of *Smarcd3*^*WT*^*-KP*^*f/f*^*C* (WT) and *Smarcd3*^*KO*^*-KP*^*f/f*^*C* (KO) mice for tumor mass^*^, tumor cell number^**^, EpCAM+, EpCAM+CD133+^**^, and EpCAM+Msi2+ cells (*n* = 15 WT, *n* = 9 KO, *n* = 5 *Msi2-GFP-KP*^*f/f*^*C* mice; two-tailed *T*-test, mean ± SEM; ^*^1 outlier, ^**^2 outliers removed, ROUT *Q* = 1%). FACS gating Supplementary Fig. 2f. **c** Secondary syngeneic transplants in *KP*^*f/f*^*C* model. **d** *Smarcd3* deletion reduces tumor burden in secondary *KP*^*f/f*^*C* transplants; *Smarcd3*^*KO*^*-KP*^*f/f*^*C* (KO) transplants have reduced EpCAM+^*^, EpCAM+CD133+ and EpCAM+Msi2+ cells (cells were isolated from *n* = 4 primary tumors/genotype and transplanted into *n* = 2–4 recipients per cohort, see Source Data File, ^*^1 outlier removed, ROUT *Q* = 1%; 2-way ANOVA, mean ± SEM). FACS gating Supplementary Fig. 2f. **e** *Smarcd3* deletion improves survival in the *KP*^*f/f*^*C* model. Median survival was 13% longer for *Smarcd3*^*KO*^*-KP*^*f/f*^*C* (KO) vs. *Smarcd3*^*WT*^*-KP*^*f/f*^*C* (WT) mice (log-rank test). Median survival was 28% longer in KO mice with gemcitabine (gem) treatment (log-rank test). *Smarcd3* deletion synergized with chemotherapy; WT survival improved 13% with *Smarcd3* deletion and 4% with gem treatment while *Smarcd3* deletion plus gem drove a 34% survival benefit. **f** Inducible *Smarcd3* deletion in *KPF* transplants. *Smarcd3*^*f/f*^ mice were crossed to a dual-recombinase model (*FSF-Kras*^*G12D/+*^*; p53*^*FRT/FRT*^*; Pdx-Flp; KPF*) and global *R26-CreER*^*T2*^ line enabling inducible *Smarcd3* deletion in transplanted tumors. **g** Inducible *Smarcd3* deletion blocks the growth of established *KPF* tumors. Vehicle and tamoxifen-treated *Smarcd3*^*f/f*^*-KPF-R26-CreER*^*T2*^ (*KPF*) transplants were analyzed for total tumor cell number and tumor area* (*n* = 3–6 transplants/tumor, see Source Data File; *2 outliers removed, ROUT *Q* = 1%; 2-way ANOVA, mean ± SEM). **h** Inducible *Smarcd3* deletion in autochthonous *KPF* mice. *Smarcd3*^*f/f*^ mice were crossed to a dual-recombinase model (*FSF-Kras*^*G12D/+*^*; p53*^*FRT/FRT*^*; Pdx-Flp; KPF*) and global *R26-CreER*^*T2*^ line, enabling inducible global *Smarcd3* deletion in autochthonous tumors. **i** Inducible *Smarcd3* deletion reduces tumor content in autochthonous *KPF* mice. Vehicle and tamoxifen-treated *Smarcd3*^*f/f*^*-KPF-R26-CreER*^*T2*^ (KPF) tumors were analyzed. Total tumor and EpCAM+ tumor cell numbers were reduced (*n* = 5 WT (Cre−) and *n* = 4 KO (Cre+) *KPF* mice; two-tailed *T*-test, mean ± SEM). FACS gating Supplementary Fig. 2f. Source data are provided in the Source Data file.

To assess the function of *Smarcd3* in advanced pancreatic tumors driven by both RAS activation and p53 loss, we crossed *Smarcd3*^*f/f*^ mice into two independent autochthonous models that enabled temporally distinct deletion of *Smarcd3*, either embryonically or in adult mice. First, *Smarcd3*^*f/f*^ mice were crossed into the *KP*^*f/f*^*C* model (Fig. 2a), where *Smarcd3* is deleted synchronously with RAS activation/*p53* deletion in pancreatic progenitors embryonically^23,24^. *Smarcd3*^*KO*^-*KP*^*f/f*^*C* tumors (Supplementary Fig. 2d, e) showed a trend towards reduced EpCAM+ tumor cell content, and a 2.5-fold reduction in EpCAM+MSI2+ cancer stem cells at midpoint (7–8 weeks) (Fig. 2b, Supplementary Fig. 2f). *Smarcd3* deletion led to a greater significant loss (3-fold) in EpCAM+ tumor cells, and a 3.5-fold reduction in EpCAM+MSI2+ tumor stem cells in secondary transplants (Fig. 2c, d), suggesting that *Smarcd3* deletion reduces the self-renewal capability of established tumor cells. *Smarcd3* deletion also slightly improved median survival of mixed background *KP*^*f/f*^*C* mice in line with previous studies^7,37^ (13% survival benefit, HR = 2.471; Fig. 2e). Further, *Smarcd3* deletion provided a much greater survival benefit in the presence of low-dose chemotherapy (gemcitabine, 28% survival benefit; HR = 3.37; Fig. 2e). These results indicate that *Smarcd3* is a potential functional dependency of cancer cells in established tumors in vivo, and demonstrate that depletion of cancer stem cells by *Smarcd3* deletion can sensitize to chemotherapy.

To directly test the function of *Smarcd3* in the context of established tumors in adult mice (uncoupled from deletion at initiation) we utilized a model that allowed for genetic deletion post-tumor establishment by crossing *Smarcd3*^*f/f*^ mice into the *FSF-Kras*^*G12D/+*^*; p53*^*frt/frt*^*; Pdx-Flp* dual-recombinase model of pancreatic cancer. In this model, *Kras* mutation and *p53* deletion are driven by a pancreas-specific *Pdx-Flp* recombinase, allowing independent spatiotemporal control over *Smarcd3* deletion with CRE^38^. To induce deletion post-establishment in vivo, *Smarcd3*^*f/f*^*; FSF-Kras*^*G12D/+*^*; p53*^*frt/frt*^*; Pdx-Flp* mice were crossed to a globally expressed tamoxifen-inducible *R26-CreER*^*T2*^ CRE^39^ (Fig. 2f). End-stage *Smarcd3*^*f/f*^*-KPF-R26-CreER*^*T2*^ (*KPF*) tumor cells were transplanted subcutaneously and recipient mice were treated with tamoxifen or vehicle once tumors were established; tumor burden was then analyzed 3 weeks later (Fig. 2f). *Smarcd3* deletion (Supplementary Fig. 2g, h) led to a 2-fold reduction in total tumor mass, tumor area, and tumor cell number in tamoxifen-treated mice (Fig. 2g) even though escaper SMARCD3 re-expression could be detected in some tumors (Supplementary Fig. 2i). To assess the impact of *Smarcd3* deletion directly in the autochthonous *KPF* model, we tracked tumor emergence by palpation and enrolled CRE+ and CRE− tumor-bearing *KPF* mice into treatment with tamoxifen followed by tumor analysis three weeks later (Fig. 2h). Despite advanced tumor stage at enrollment, *Smarcd3* deletion reduced total tumor cell and EpCAM+ tumor cell number by 1.5 fold (Fig. 2i). Together, these data show that *Smarcd3* represent a functional dependency in the context of established and advanced pancreatic tumors.

### SMARCD3 knockdown blocks tumor growth in human models of pancreatic cancer

**Fig. 3:**
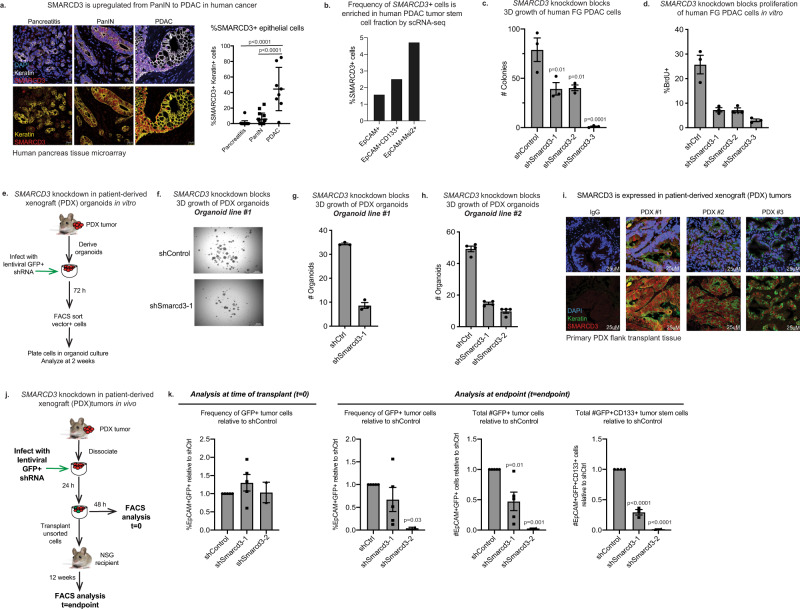
SMARCD3 knockdown blocks tumor growth in human models of pancreatic cancer. **a** SMARCD3 is upregulated from PanIN to PDAC in human cancer. Frequency of nuclear (DAPI, blue) SMARCD3+ (red) epithelial cells (pan-keratin+, white/yellow) in pancreatitis (*n* = 22), PanIN (*n* = 15), and PDAC (*n* = 8); n = 3 frames per case; ANOVA with multiple comparisons, mean ± SEM (scale bar = 25 μm). **b** The frequency of *SMARCD3*+ cells is increased in the stem cell fraction of primary human PDAC tumors by scRNA-seq^40^. *SMARCD3*+ cells were quantified within *MSI2+* and *CD133+EpCAM+* tumor stem cells relative to bulk *EpCAM+* tumor cells (see Supplementary Fig. 3a). **c** Knockdown of *SMARCD3* using shRNA blocks 3D growth of human FG PDAC cells in vitro (representative, *n* = 4 biological replicates at *n* = 3; ANOVA with multiple comparisons, mean ± SEM). **d** Knockdown of *SMARCD3* using shRNA blocks proliferation of human FG PDAC cells in vitro (*n* = 1 biological replicate at *n* = 3, mean ± SEM). **e** Transduction of patient-derived organoids with *SMARCD3* shRNA in vitro. **f** *SMARCD3* knockdown blocks growth of patient-derived PDAC organoids in vitro (representative, *n* = 1 organoid line at *n* = 3 replicates, scale bar = 1 mm). **g** *SMARCD3* knockdown blocks growth of patient-derived PDAC organoids in vitro; organoid line #1 1 (*n* = 1 biological replicate at *n* = 3; mean ± SEM). **h** *SMARCD3* knockdown blocks growth of patient-derived PDAC organoids in vitro; organoid line #2 (*n* = 1 biological replicate at *n* = 4; mean ± SEM). **i** Patient-derived xenograft (PDX) tumors express SMARCD3. Three PDX tumors were stained for nuclear (DAPI, blue) SMARCD3 (red) within the epithelium (pan-keratin, green) (scale bar = 25 μm). **j** Transduction and transplant of patient-derived xenograft tumor cells. **k** *SMARCD3* knockdown blocks in vivo growth of patient-derived xenograft PDAC tumors. Tumors were analyzed for GFP (shRNA vector), EpCAM, and CD133 expression. Despite equivalent transduction at t = 0 (left), frequency of GFP+EpCAM+ tumor cells (middle left), the number of GFP+EpCAM+ tumor cells (middle right), and the number of GFP+CD133+EpCAM+ tumor stem cells (right) were reduced by *SMARCD3* knockdown (ANOVA with multiple comparisons, mean ± SEM). FACS plots Supplementary Fig. 3g. Source data for all studies are provided in the Source Data file.

Although genetically engineered mouse models (GEMMs) are useful models that are representative of human disease, PDAC patient tumors are diverse and exhibit more complex mutational landscapes. While SMARCD3 was rarely expressed in benign inflamed tissue (pancreatitis), the frequency of nuclear SMARCD3+ epithelial cells rose in PanIN and, to a greater degree, in PDAC in a human tissue microarray (Fig. 3a). In addition, the frequency of *SMARCD3* expression was enriched within *PROM1*+ (CD133+ ) and *MSI2*+ tumor cells (1.5-fold and 3-fold, respectively) in primary human PDAC tumors in a published single-cell RNA-seq dataset^40^ (Fig. 3b, Supplementary Fig. 3a), supporting the data from genetic models (Fig. 1a, f, Supplementary Fig. 1c, d). To determine if SMARCD3 expression was associated with common PDAC driver events, we stained a clinically annotated tissue microarray for SMARCD3 expression by immunohistochemistry (IHC) (Supplementary Fig. 3b). SMARCD3 expression was, as a trend, most closely associated with *KRAS* mutation in human PDAC; SMARCD3 was expressed in 58% of *KRAS*^*MUTANT*^ tumors and only 17% of *KRAS*^*WT*^ tumors (Supplementary Fig. 3c). SMARCD3 was also expressed in the context of mutant *Kras* alone in the *KC* GEMM (Supplementary Fig. 3d), and *Kras* knockdown reduced *Smarcd3* expression by 5-fold in *KP*^*f/f*^*C* cells in vitro (Supplementary Fig. 3e). These data indicate that SMARCD3 is upregulated in human PDAC and suggest an upstream role for RAS in the regulation of SMARCD3 in cancer.

To test whether SMARCD3 is a functional dependency in human pancreatic tumors, we knocked down *SMARCD3* in the human FG PDAC cell line (Supplementary Fig. 3f). shRNA-mediated *SMARCD3* knockdown markedly inhibited the 3D growth of FG cells (Fig. 3c), reducing proliferation by 5-fold (Fig. 3d). Knockdown of *SMARCD3* also reduced the 3D growth of two independent patient-derived organoid lines in vitro by greater than 3-fold (Fig. 3e–h). To extend these findings in vivo, we knocked down *SMARCD3* in three independent SMARCD3+ patient-derived xenograft (PDX) tumors (Fig. 3i, j). PDX tumors were dissociated and infected with GFP-tagged lentiviral shRNA in vitro, and then re-transplanted subcutaneously in NSG mice (Fig. 3j). While each PDX sample was transduced equivalently at *t* = 0 (Fig. 3k, left and Supplementary Fig. 3g), the relative frequency and total number of GFP + EpCAM+ tumor cells were reduced by 2–50-fold in sh*Smarcd3* tumors at endpoint (Fig. 3k, middle and Supplementary Fig. 3g). Further, the total number of CD133+ stem cells within the GFP+EpCAM+ tumor fraction was reduced by up to 100-fold in sh*Smarcd3-*treated tumors relative to sh*Control* (Fig. 3k, right). These data indicate a strong dependence of patient-derived primary PDAC tumor cells in general, and the most therapy-resistant CD133+ stem cells in particular, on SMARCD3 for in vivo growth and propagation.

### SMARCD3 regulates the epigenetic landscape and BAF complex binding at FOXA1 binding sites in mouse pancreatic cancer cells

**Fig. 4:**
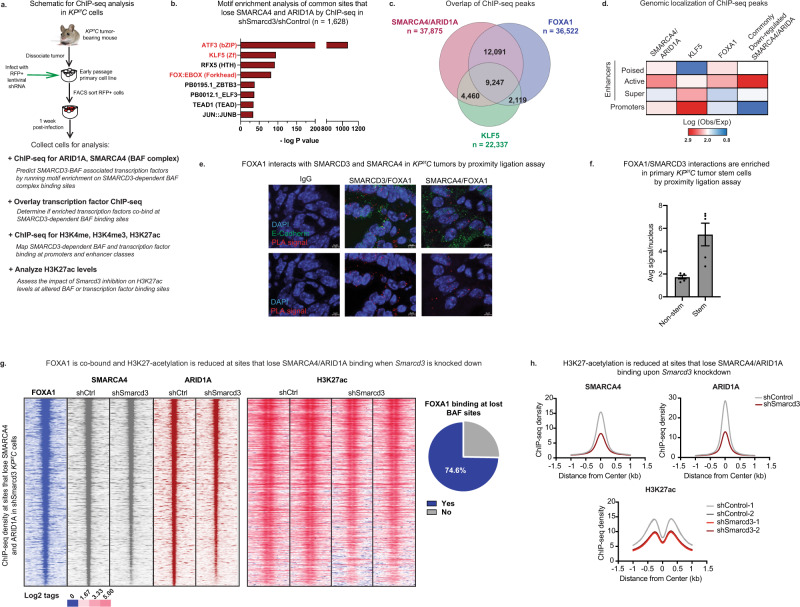
SMARCD3 regulates the epigenetic landscape and BAF complex binding at FOXA1 binding sites in mouse pancreatic cancer cells. **a** Schematic for ChIP-seq analysis in *KP*^*f/f*^*C* cells. **b** Motif enrichment on sites that lose SMARCA4/ARID1A binding upon *Smarcd3* is knockdown. Motif enrichment (cumulative binomial distribution) on commonly down-regulated SMARCA4/ARID1A binding sites by ChIP-seq shows that commonly lost sites are enriched for ATF3 (AP-1), KLF5, and FOX (FOXA1) motifs. **c** FOXA1 and KLF5 binding sites overlap with SMARCA4/ARID1A binding sites in *KP*^*f/f*^*C* cells. FOXA1 and KLF5 ChIP-seq in *KP*^*f/f*^*C* cells were overlaid with SMARCA4/ARID1A ChIP-seq to identify overlapping binding sites. **d** SMARCA4, ARID1A, and FOXA1 binding are enriched at active enhancers. ChIP-seq for H3K27ac, H3K4me, and H3K4me3 allowed mapping of cis-regulatory genomic elements (poised, active, and super-enhancers as well as promoters). SMARCA4/ARID1A co-bound sites and FOXA1 are most enriched at active enhancers; KLF5 is enriched at promoters. Common sites that lose SMARCA4/ARID1A binding upon *Smarcd3* knockdown are significantly enriched at active enhancers. **e** FOXA1 interacts with SMARCD3 and SMARCA4 in vivo. Proximity ligation assay (PLA) with antibodies against FOXA1, SMARCD3, and SMARCA4 showed positive PLA (red) in the nuclei (DAPI, blue) of *KP*^*f/f*^*C* tumor cells (E-Cadherin, green), representing associations between FOXA1/SMARCD3, and FOXA1/SMARCA4 in mouse pancreatic tumor tissue (representative, *n* = 2 mice, *n* = 5 frames/tumor, mean ± SEM, scale bar = 5 μm; see Supplementary Fig. 4c, d). **f** FOXA1/SMARCD3 interactions are enriched in primary *KP*^*f/f*^*C* stem cells. Proximity ligation assay with antibodies against FOXA1 and SMARCD3 showed positive PLA signals were enriched in CD133+ tumor stem cells (*n* = 1 mouse, *n* = 5 frames/tumor, mean ± SEM). Source data are provided in the Source Data file. **g** FOXA1 is co-bound and H3K27-acetylation is reduced at sites that lose SMARCA4/ARID1A binding upon *Smarcd3* knockdown. SMARCA4 and ARID1A ChIP-seq density at sites commonly lost when *Smarcd3* is knocked down overlap with FOXA1 binding (left); H3K27-acetylation is reduced (middle) and FOXA1 is co-bound at 75% of sites (right) that commonly lose BAF binding upon *Smarcd3* knockdown. **h** H3K27-acetylation is reduced at sites that lose SMARCA4/ARID1A binding upon *Smarcd3* knockdown. Sites where SMARCA4/ARID1A binding is lost (upper row) show reduced H3K27-acetylation (bottom row).

As a subunit of a chromatin-modifying complex, SMARCD3 may control tumor cell function by regulating SWI/SNF binding and the epigenetic landscape. SWI/SNF complexes exist as three variants (BAF, PBAF, ncBAF)^41–44^; of these, SMARCD3 was predominantly incorporated into the more abundant BAF complex and to some extent PBAF in *KP*^*f/f*^*C* cells (Supplementary Fig. 4a, b). Thus, we focused on defining SMARCD3-dependent changes in BAF complex binding using chromatin immunoprecipitation followed by genome-wide sequencing (ChIP-seq) with antibodies against the core ATP-ase SMARCA4 and BAF-specific ARID1A (Fig. 4a). *Smarcd3* loss reduced both SMARCA4 and ARID1A binding at 1,628 common sites (Fold change 1.5, Poisson *p* value = 0.05). Motif-enrichment analysis on these SMARCD3-dependent BAF binding sites revealed significant enrichment for KLF5 and FOXA1 motifs, as well as AP-1 (ATF3), which served as a control^45^ (Fig. 4b). ChIP-seq for FOXA1 and KLF5 in *KP*^*f/f*^*C* cells confirmed that FOXA1 and KLF5 were indeed co-bound with SMARCA4 and ARID1A at 56% and 36% of sites, respectively, suggesting a possible association between these factors and the SMARCD3-containing BAF complex (Fig. 4c).

SWI/SNF complexes typically regulate cell fate by binding to cis-regulatory elements of the genome, including promoters and enhancers. Using ChIP-seq for H3K4me, H3K4me3, and H3K27ac histone modifications that can be used to distinguish cis-regulatory elements (Fig. 4a)^46–48^, we found that SMARCA4 and ARID1A co-bound sites, and downregulated co-bound sites in particular, were preferentially enriched at active enhancers (Fig. 4d), suggesting SMARCD3 loss differentially impacted BAF complex binding at enhancers relative to promoters. While KLF5 binding was most enriched at promoters, FOXA1 binding was enriched at active enhancers, suggesting that FOXA1 may be the more relevant partner for SMARCD3 activity. Consistent with this, proximity ligation and co-immunoprecipitation showed FOXA1 interacting with both SMARCD3 and SMARCA4 in *KP*^*f/f*^*C* tumor cells (Fig. 4e, Supplementary Fig. 4c–e); this interaction was enriched in primary *KP*^*f/f*^*C* stem cells (Fig. 4f). Further, FOXA1 was co-bound at 75% of common SMARCD3-dependent BAF binding sites (Fig. 4g); these results support a collaboration between the SMARCD3-containing BAF complex and FOXA1 in pancreatic cancer cells. Supporting a role for SMARCD3 and FOXA1 in cancer stem cells, SMARCD3/FOXA1 interactions were enriched within the nuclei of primary CD133+ mouse tumor cells and *Foxa1* knockdown severely reduced sphere formation in *KP*^*f/f*^*C* cells (Supplementary Fig. 4f). *Smarcd3* knockdown also led to reduced H3K27ac at sites that lost SMARCA4/ARID1A binding (Fig. 4g, h), predicting reduced transcriptional activity at these conserved SMARCD3-dependent BAF complex binding sites.

### SMARCD3 regulates transcriptional networks implicated in lipid metabolism

**Fig. 5:**
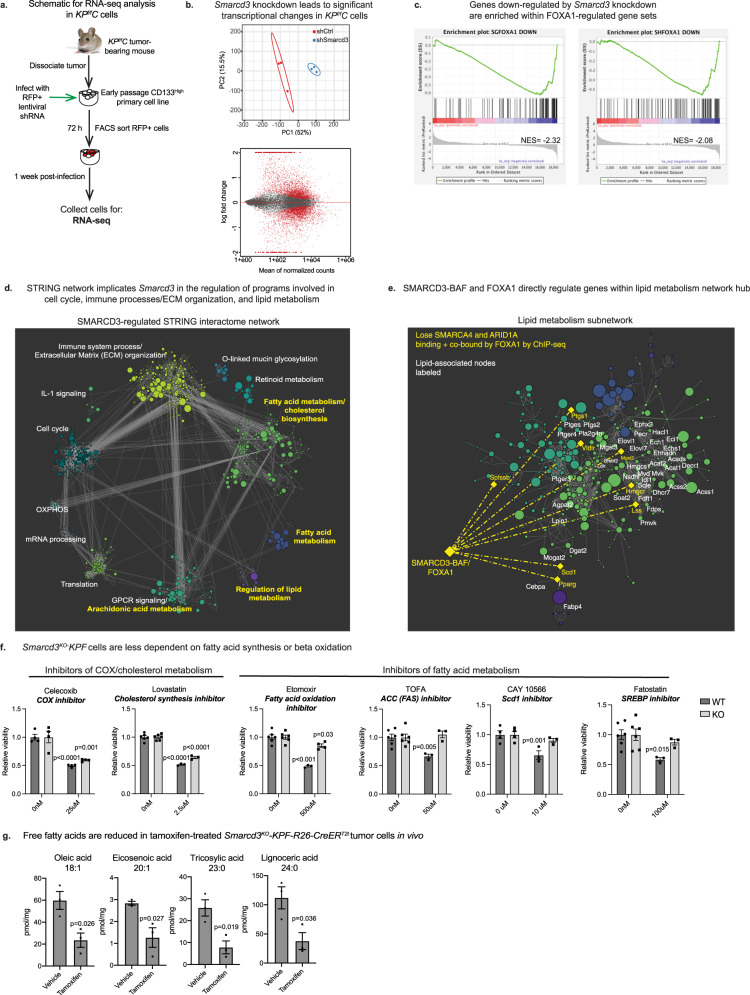
SMARCD3 regulates transcriptional networks implicated in lipid metabolism. **a** Schematic for RNA-seq analysis in *KP*^*f/f*^*C* cells. **b** *Smarcd3* knockdown impacts transcription in *KP*^*f/f*^*C* cells. PCA plot (top) demonstrates the clustering of shControl (red) and sh*Smarcd3* (blue) replicates by RNA-seq. MA plot (bottom) of differential gene expression by RNA-seq; normalized counts are plotted against log fold change in expression. Differentially expressed genes are in red. **c** Genes down-regulated by *Smarcd3* knockdown are enriched within FOXA1-regulated gene sets. Gene set enrichment analysis (GSEA) of RNA-seq data revealed enrichment for FOXA1-regulated gene sets within genes down-regulated by *Smarcd3* knockdown (Supplementary Fig. 5a). **d** STRING network implicates *Smarcd3* in the regulation of programs involved in the cell cycle, immune processes, extracellular matrix organization, and lipid metabolism. Down-regulated genes (padj < 0.05, log(fold change)<−0.35) were used to map the SMARCD3-dependent network within the STRING interactome (node size scaled to log(fold change)). A clustering algorithm was applied to the network to generate 12 programmatic hubs; STRING functional enrichment was used to identify enriched annotations for each hub (hubs with lipid-related annotations are labeled yellow). **e** SMARCD3-BAF and FOXA1 directly regulate genes within the lipid metabolism network hub. Lipid-associated network hubs were merged and nodes with lipid-metabolic functions were labeled. We identified potential direct targets of SMARCD3-BAF/FOXA1 (yellow label) as genes bound by FOXA1 that lose BAF (SMARCA4/ARID1A) binding by ChIP-seq when *Smarcd3* is knocked down. **f** *Smarcd3*^*KO*^*-KPF* cells are less dependent on fatty acid synthesis and beta-oxidation. Curated screen of metabolic inhibitors conducted in *Smarcd3*^*WT*^ and *Smarcd3*^*KO*^*-KPF* cells. Primary tumor cell lines were derived from (Cre−) *Smarcd3*^*f/f*^*-KPF* tumors. *Smarcd3* deletion was driven by the delivery of adenoviral GFP (WT) or Cre (KO); transduced cells were treated with inhibitors for 72 h in sphere-forming conditions and assessed for viability (representative, *n* = 3 biological replicates with *n* = 4 technical replicates per experiment; 2-way ANOVA with multiple comparisons, mean ± SEM). **g** Free fatty acids are reduced in tamoxifen-treated *Smarcd3*^*f/f*^*-KPF-R26-CreER*^*T2*^ tumors. Vehicle or tamoxifen-treated *Smarcd3*^*f/f*^*-KPF-R26-CreER*^*T2*^ EpCAM+ tumor cells were sorted for free fatty acid analysis by GC-MS (*n* = 3, two-tailed *T*-test, mean ± SEM; see Supplementary Fig. 5c, d). All Source data are provided in the Source Data file.

We analyzed the functional consequence of these epigenetic changes on gene expression using RNA-seq analysis of sh*Smarcd3*-treated *KP*^*f/f*^*C* cells (Fig. 5a)*. Smarcd3* knockdown drove the differential expression of over a thousand genes (Fig. 5b), with these changes overlapping significantly with FOXA1-regulated gene sets^49^, supporting a co-regulatory function for FOXA1 and SMARCD3 (Fig. 5c, Supplementary Fig. 5a). A high-confidence STRING network of down-regulated genes (Fig. 5d; nodes colored by cluster, node size scaled to logFC) identified 12 SMARCD3-regulated transcriptional hubs enriched for diverse functions including glycosylation, extracellular matrix organization and immune signaling (Fig. 5d, Supplementary Data 1). Strikingly, four of these hubs converged on lipid metabolism (Fig. 5d in yellow, Fig. 5e), which was also the most significantly enriched term in the Gene Ontology analysis of the RNA-seq dataset (Supplementary Fig. 5b). These lipid metabolism hubs encompassed functions in arachidonic acid metabolism, fatty acid metabolism, cholesterol biosynthesis, and metabolic regulation. As lipid metabolism has emerged as an important feature of aggressive cancer stem cell populations^20^, we further focused on this functional program.

Within lipid-associated network hubs (Fig. 5e), SMARCD3-regulated genes were involved at almost every level of lipid homeostasis. *Smarcd3* loss downregulated lipid transport and storage genes, as well as major transcriptional regulators of lipid metabolism (Supplementary Table 1). Further, *Smarcd3* knockdown drove down the expression of core enzymes involved in the metabolism of lipid families with known functions in cancer: cholesterol, prostaglandins, and fatty acids (Supplementary Table 1). Both cholesterol and fatty acid metabolism are enriched in cancer stem cells and have been associated with stem cell signaling and therapy resistance in many cancers^20,50,51^, indicating that SMARCD3 may regulate stem cell-enriched metabolic pathways. Several core genes within the lipid subnetwork such as *Pparg, Scd1, Hmgcr, Ptgs1*, and *Vldlr* were directly bound by SMARCD3-BAF and FOXA1, highlighting a direct coordinated role for SMARCD3-BAF and FOXA1 in the regulation of lipid homeostasis.

We next used a curated metabolic screen to test whether transcriptional changes in lipid pathways reflected functional shifts and found that while *Smarcd3*^*KO*^*-KPF* cells retained dependence on cholesterol metabolism (Lovastatin) and prostaglandin synthesis or COX (Celexcoxib), they had lost sensitivity to inhibitors of fatty acid synthesis (TOFA, CAY10566, Fatostatin) and beta oxidation (Etomoxir) in vitro (Fig. 5f). Further, tamoxifen-mediated *Smarcd3* deletion led to a ~3-fold drop in total free fatty acid content in EpCAM+ *Smarcd3*^*f/f*^*-KPF-R26-CreER*^*T2*^ tumor cells in vivo, as determined by gas chromatography–mass spectrometry (GC–MS) (Supplementary Fig. 5c). Of all downregulated fatty acid species (Supplementary Fig. 5d), the most significant were the monounsaturated fatty acids oleic acid (C18:1) and eicosenoic acid (C20:1), and the long chain saturated fatty acids tricosylic (C23:0) and lignoceric acid (C24:0) (Fig. 5g), which can contribute to the synthesis of complex lipids and play a role in signaling and survival in cancer cells^52^. Collectively, these results demonstrate that SMARCD3-BAF, in concert with FOXA1, is an important regulator of fatty acid metabolism, and draws a link between SWI/SNF and stem cell-enriched metabolic programs in pancreatic cancer.

## Discussion

Despite clinical advances in many cancers, pancreatic cancer mortality remains high, driven by early metastasis and therapy resistance^3^ which can be attributed in part to cancer stem cells, subpopulations within the tumor bulk enriched for developmental signals and self-renewal^5–9^. These therapy-resistant cells are epigenetically unique^8^, suggesting that they may exploit developmental epigenetic mechanisms to maintain a stem cell state and drive relapse. Our work here has led to the identification of SMARCD3 as a potential stem cell-inclusive functional dependency in pancreatic cancer. SMARCD3 is a subunit of SWI/SNF, a nucleosome remodeling complex with core functions in development and cancer. Given the potential for SWI/SNF to coordinate a broad range of cell-type specific functions, targeting cancer-specific SWI/SNF activity is an appealing therapeutic paradigm^17,18^. To this end, several studies have shown that SWI/SNF-mutant cancers can be successfully treated by inhibiting residual synthetic-lethal complex subunits^18^. However, although over 20% of cancers are SWI/SNF-mutant^31^, the mechanisms by which dysregulated SWI/SNF activity contributes to tumor heterogeneity and disease progression in the remaining 80% of cancers remains relatively unexplored, and defining these could have far-reaching impacts on therapy^17,18^. Further, the role of SWI/SNF in the establishment and propagation of therapy-resistant cancer stem cells remains largely understudied, and our work provides an important complement to emerging studies showing that SWI/SNF ATP-ase SMARCA4 supports stem function in glioma^13,14^ and in leukemia^16^.

Here, we show that SMARCD3 is enriched in the stem cell fraction of pancreatic tumors, and is a potential functional dependency of established cancer cells in vivo. Although SMARCD3 expression and function may not be exclusive to cancer stem cells alone, we found that *Smarcd3* was a stem-inclusive dependency whose perturbation was sufficient to block the growth of pancreatic cancer stem cells in vivo. Using a diverse set of conditional genetic models, we also identified stage-specific roles for *Smarcd3* in pancreas cancer. Unlike deletion of *Smarca4*^53,54^, *Smarcd3* deletion in the context of *Kras* mutation alone only slightly promoted the emergence of cystic lesions from pancreatic progenitors but had no significant impact on PanIN 1A development in any model of initiation. Thus, SMARCD3 may not have a robust impact on early tumor initiation, although its restricted ductal expression in the normal mouse pancreas (as opposed to the ubiquitous expression of SMARCA4) suggests a potential role in ductal fate (Supplementary Fig. 5e). This raises the possibility that SMARCD3 may enable cell-type-specific functions of SMARCA4 to enforce cell fate in normal duct cells. Similarly, the fact that SMARCD3 is elevated from PanIN to PDAC suggests that it may be required to support ductal fate later in disease progression, and serve as an important enabler of SMARCA4 function in cancer.

Because loss-of-function alterations in *SMARCD3* have not been identified in cancer, it is unlikely that its deletion significantly drives tumorigenesis in human disease. Instead, amplifications in *SMARCD3* have been detected^19^ and we found that SMARCD3 expression increased most robustly from PanIN to PDAC in human tissues, supporting a more oncogenic role for SMARCD3 in cancer progression. In support of this, we found that genetic *Smarcd3* deletion in the *KP*^*f/f*^*C* model blocked the growth of secondary transplants, synergized with chemotherapy, and improved survival. Further, using the dual-recombinase *KPF* model, we directly demonstrated that *Smarcd3* deletion impaired the growth of established tumors in both the transplant setting and the autochthonous model. Consistent with this, SMARCD3 was required for the propagation of patient-derived xenografts in vivo, providing strong evidence that *Smarcd3* is required for advanced cancer growth. This aligns with a pro-tumorigenic function for SMARCD3 identified in breast cancer^55^ and stands in contrast to *Smarcb1*^56^ or *Arid1a*^57^, which serve as tumor suppressors in established tumors. Our work shows that SWI/SNF function is dependent on the cellular context, highlighting the importance of testing genetic SWI/SNF deletion in the appropriate context in GEMMs and demonstrating the utility of dual-recombinase models for investigating chromatin remodeler function in cancer. In contrast to our findings that *Smarcd3* is a potential cancer dependency, the cancer dependency DepMap database shows that *SMARCD3* is an enrichment in cancer cell lines, further suggesting that SMARCD3 function may be context-dependent. While DepMap explores gene function in 2D cancer cell lines, our studies have used both primary mouse cell lines and more physiologically relevant patient-derived organoids in 3D culture systems as well as *five* distinct genetically engineered models and patient-derived xenografts. It should be noted that DepMap does not accurately predict the function of several other well-known cancer-associated genes. *PROM1* (CD133) is an enrichment within PDAC lines despite its function in cancer invasion^58^. DepMap also predicts an opposing function for SWI/SNF component ARID1A, which appears as a slight dependency in contrast to its apparent function as a tumor suppressor in PDAC^57^. Although encompassing broad cell lines, this type of 2D functional screening may miss or underestimate the impact of genes playing a role in invasiveness, 3D/in vivo growth, or stemness. Further, recent work^59^ has identified *Smarcd3* as a top hit in a screen for epigenetic regulators that bypass KRAS dependency. This indicates that SMARCD3 overexpression is sufficient to drive tumor growth even in the absence of this critical oncogene, supporting a pro-tumorigenic function for *Smarcd3* in PDAC.

As a SWI/SNF subunit, SMARCD3 can exert broad regulatory control over epigenetic and transcriptional programs, likely by scaffolding transcription factors^60^. Integrating RNA-seq and ChIP-seq via network analysis we found that *Smarcd3* knockdown drove losses in BAF complex binding and H3K27-acetylation at active enhancers co-bound by FOXA1. FOXA1 was directly associated with both SMARCD3 and SMARCA4 in vivo, suggesting that SMARCD3 coordinates FOXA1/BAF activity at a subset of sites, controlling downstream transcriptional programs with diverse functions including extracellular matrix organization, glycosylation, and immune signaling (Supplementary Data 1). The regulation of these programs suggests a putative role for SMARCD3 in orchestrating interactions between pancreatic cancer cells and the microenvironment. The regulation of prostaglandin synthesis by SMARCD3 could also impact inflammation in the tumor microenvironment. SWI/SNF mutational status can determine immunotherapy response in some cancers^61^, so connections between SMARCD3 and the tumor microenvironment may be a clinically relevant avenue for future study.

A key finding in our work is the discovery that SMARCD3 controls the landscape of lipid metabolism in pancreatic cancer cells. While genes involved in cholesterol, prostaglandin, and fatty acid synthesis and beta-oxidation were all downregulated by *Smarcd3* knockdown, *Smarcd3*^*KO*^ cells specifically lost dependence on fatty acid pathways and exhibited reduced fatty acid content in vivo. These results link SMARCD3 and fatty acid metabolism, which has been associated with therapy resistance in cancer^20^. Though SWI/SNF has been shown to regulate metabolism^62^, this work connects SWI/SNF and the regulation of cancer lipid metabolism through SMARCD3 and FOXA1. Given the emerging role of fatty acid metabolism in therapy-resistant cancer cells^20^, these results position SMARCD3 as an important regulator of stem cell-enriched metabolic programs. The role of SMARCD3 in metabolic regulation is also particularly interesting given its nutrient-sensing function in normal tissues^63^; it is possible that SMARCD3 may similarly act as a metabolic sensor in cancer. In support of this, we found that *Smarcd3* expression and protein stability were sensitive to glucose in vitro (Supplementary Fig. 5f–h), suggesting that SMARCD3 may have the potential to integrate SWI/SNF and transcription factor activity to enable epigenetic adaptation to the metabolic environment. The mechanisms by which SMARCD3 may sense metabolic status could provide insight into the role of SWI/SNF in regulating metabolic plasticity in cancer. Collectively, our results position SMARCD3 as an oncogenic SWI/SNF subunit that could drive important metabolic functions in aggressive cancer cells and serve as an effective target for new therapies.

## Methods

### Lead contact

Further information and requests for resources and reagents should be directed to and will be fulfilled by the Lead Contact, Dr. Tannishtha Reya (tr2726@cumc.columbia.edu).

### Ethical approval

All animal experiments were performed according to protocols approved by the University of California San Diego Institutional Animal Care and Use Committee (protocol S12100). Patient-derived xenograft (PDX) tumors and organoids were derived from originally consented PDAC patients in accordance with the Declaration of Helsinki and use was approved by UCSD’s IRB; written consent was obtained. Samples were de-identified and no further information on patient status, treatment, or otherwise, is available. The creation and use of the TMAs were reviewed and approved by the Ethics Committee at the University of Athens, Greece, and the University of Bern, Switzerland, and included written informed consent from the patients or their living relatives.

### Mice

Mice were pathogen-free, and bred and maintained in the animal care facilities at the University of California San Diego; all animals were maintained as mixed background. Animals had access to food (Teklad # 5053) and water ad libitum and were group-housed in ventilated cages under controlled temperature and humidity with a 12-h light–dark cycle. No sexual dimorphism was noted, therefore, males and females of each strain were equally used for experiments and both sexes are represented in all data sets. Littermates of the same sex were randomized into experimental groups when possible. Only female NOD/SCID and NSG mice were used, all at ages 6–8 weeks. The exact strain, age, and sex of all mice used are outlined in Supplementary Data 2. Experiments involving subcutaneous injections were monitored and sacrificed before exceeding the UCSD Institutional Animal Care and Use Committee limit of 1.5 cm. Mice were euthanized using CO_2_ as the primary method, followed by cervical dislocation, decapitation, or tissue removal as a secondary method to ensure death in accordance with protocols approved by the University of California San Diego Institutional Animal Care and Use Committee.

The *LSL-Kras G12D* (*Kras*^*G12D/+*^) mouse, B6.129S4-Kras^tm4Tyj/J^ (Stock No. 008179), *p53*^*flox/flox*^ (*p53*^*f/f*^) mouse, B6.129P2- Trp53^tm1Brn/J^ (Stock No. 008462), *R26-CreER*^*T2*^ mouse, B6.129-*Gt(ROSA)26Sor*^*tm1(cre/ERT2)Tyj*^/J (Stock No. 008463), *Ptf1a*^*CRE-ERTM*^, Ptf1a^tm2(cre/ESR1)Cvw^/J (Stock No. 019378), and the *Sox9-CreER*^*T2*^, Tg(Sox9-cre/ERT2)1Msan/J (Stock No. 018829) were purchased from The Jackson Laboratory. *Msi2*^*eGFP/+*^ (Msi2-GFP) reporter mice were generated as previously described^7^; all of the reporter mice used in experiments were heterozygous for the Msi2 allele. Dr. Chris Wright provided *p48-Cre* (*Ptf1a-Cre*) mice as previously described^21^. *LSL-R172H* mutant *p53* (p53^R172H*/+*^), Trp53^R172H^ mice were provided by Dr. Tyler Jacks as previously described^64^ (JAX Stock No. 008183). Dr. Benoit Bruneau generated *Smarcd3*^*f/f*^ mice as previously described^35^; mice were provided by Dr. Lorenzo Puri. Dr. Dieter Saur provided *Pdx-FlpO*^*KI*^ (*Pdx-Flp*), *p53*^*frt/frt*^ and *FSF-Kras*^*G12D/+*^ mice as previously described^38^. Immune compromised NOD/SCID (NOD.CB17-Prkdc^scid^/J, Stock No. 001303) and NSG (NOD.Cg-Prkdc^scid^IL2rg^tm1Wji^/SzJ, Stock No. 005557) mice were purchased from The Jackson Laboratory.

### Tissue dissociation and FACS analysis

Mouse pancreatic tumors from mid-point *KP*^*f/f*^*C* mice, syngeneic secondary *KP*^*f/f*^*C* transplants, *KPF* and *KPF-R26-CreER*^*T2*^ transplants were dissociated and analyzed by FACS as follows. Tumors were washed in MEM cut into 1–2 mm pieces, and collected in 10 ml Gey’s balanced salt solution, 5 mg Collagenase P, 2 mg Pronase, and 0.2 µg DNAse I. Samples were incubated for 15 min at 37 °C, then pipetted up and down 10 times and returned to 37 °C. After 15 more minutes, samples were pipetted up and down 5 times, then passed through a 100 µm nylon mesh (Corning). Red blood cells were lysed using RBC lysis buffer and the remaining tumor cells were washed and resuspended in HBSS containing 2.5% FBS and 2 mM EDTA (HSCB) for FACS analysis, and sorting (carried out on a FACSAria III machine, FACS Diva v6.1.3, Becton Dickinson). Data were analyzed with FlowJo software v.10.5.3 (Tree Star). Antibodies and dilutions for FACS analysis are listed in Supplementary Table 2. Propidium-iodide (Life Technologies) was used to stain for dead cells. MSI2 expression was assessed by GFP expression in *Msi2-GFP-KP*^*f/f*^*C* mice.

Patient-derived xenograft tumors were dissociated to single cells by adapting from Tuveson lab organoid protocols^65,66^. Tumors were washed in MEM and cut into 1–2 mm pieces, which were collected in 10 ml Gey’s balanced salt solution, 5 mg Collagenase P, 0.2 µg DNAse I, and 10.5 μM Rho Kinase inhibitor. Samples were incubated for 10 min at 37 °C, then pipetted up and down 10 times and returned to 37 °C. After 10 more minutes, samples were pipetted up and down 5 times, then passed through a 100 µm nylon mesh (Corning). Red blood cells were lysed using RBC lysis buffer and the remaining tumor cells were washed and resuspended in HSCB for staining. Antibodies and dilutions for FACS analysis are listed in Supplementary Table 2.

### Cell lines

Mouse primary pancreatic cancer cell lines were established from end-stage mice by dissociating tumors as above and plating in 1× DMEM containing 10% FBS, 1× pen/strep, and 1× non-essential amino acids (NEAA). At the first passage, cells were resuspended in HSCB and stained with anti-EpCAM APC. EpCAM-APC+ tumor cells were sorted and re-plated. Cells were analyzed by FACS again at the second passage for markers of blood cells (CD45-PeCy7), endothelial cells (CD31-PE), and fibroblasts (PDGFR-BV421). Cell lines were derived from both female and male mice; both sexes are equally represented in the studies outlined below. Specific reagent information is provided in Supplementary Table 3.

FG human pancreatic cancer cells (COLO-357) were provided by Dr. Andrew Lowy; these cells were derived from a PDAC metastasis and have been validated^67^ and authenticated by short tandem repeat (STR) profiling. FG cells were maintained in 2D culture in 1× DMEM containing 10% FBS, 1× pen/strep, and 1x× NEAA. Cells were tested for the presence of mycoplasma and verified to be negative.

### Patient-derived xenografts and organoids

Organoid lines were derived by dissociating PDX tumors as described above. Cells were resuspended in Matrigel, and plated in 24-well plates in 25 uL Matrigel domes. Domes were covered in human organoid growth media, and maintained as previously described^65,66^. Growth media contained Advanced DMEM/F12, 10 mM HEPES (pH 7.2–7.5), 1X GlutaMAX, 100 μg/mL primocin, 50% Wnt3a conditioned media, 10% R-Spondin1-conditioned media, 1X-B27 supplement, 10 mM nicotinamide, 1.25 mM N-acetyl cysteine, 100 ng/mL murine noggin, 50 ng/mL human-EGF, 100 ng/mL human-FGF, 10 nM human gastrin, 500 nM A-83-01. Specific reagent information is provided in Supplementary Table 3.

### Patient cohort for PDAC tissue microarray

The PDAC patient cohort and corresponding TMAs used for SMARCD3 immunohistochemical staining and analysis have been reported previously^68^. Three TMAs with 0.6 mm core size were constructed with samples from the tumor center and invasive front (mean number of spots per patient: 10.5, range: 2–27). Tumor samples from 116 patients (53 females and 63 males; mean age: 64.1 years, range: 34–84 years) with a diagnosis of PDAC were included. 99 of these patients received some form of chemotherapy; 14 received radiotherapy.

### Method details

#### Sphere formation assay

Sphere formation assays were modified from Rovira et al. 2010^25^. *KP*^*f/f*^*C* cell lines were infected with lentiviral RFP-tagged shRNAs; similarly, *KPF* cell lines were infected with adenoviral GFP (adGFP) or GFP-tagged Cre (adCre). Transduced cells were sorted 72 h after transduction. 350 cells/well were suspended in sphere media: 100 µl DMEM F-12, 1x B-27, 3% FBS, 100 µM Β-mercaptoethanol, 1x NEAA, 1x N2, 20 ng/ml EGF, and 20 ng/ml bFGF2. Cells were plated in 96-well ultra-low adhesion plates and incubated at 37 °C for 7 days. For imaging, 10,000 cells were plated in 500 μL sphere media in a 24-well ultra-low attachment plate for one week. Images were acquired on a Zeiss Axiovert 40 CFL. For metabolic inhibitor studies, *KPF* cells were plated as described above in 90 μL/well media. The day after plating, 10 μL inhibitor or vehicle (DMSO) was added to cells. Viability was assessed 72 h later using the 3D CellTiterGlo assay. Inhibitors tested included celecoxib, lovastatin, etomoxir, TOFA, CAY10566, and Fatostatin. Specific reagent information is provided in Supplementary Table 3.

#### Matrigel colony assay

*KPC* cells were transduced as above. FG cells were infected with GFP-tagged shRNAs and sorted after 72 h. 500 *KPC* or FG cells were resuspended in 50 µl sphere media, mixed 1:1 with 50 μL Matrigel and plated in 96-wellplates (Costar). After incubation at 37 °C for 5 min, 50 µl sphere media was placed over the Matrigel layer. Colonies were counted 7 days later.

#### Organoid culture assays

Organoid lines were derived and maintained as above. For shRNA studies, organoids were isolated from Matrigel using Cell Recovery Solution, then dissociated to single cells with TrypLE Express with 25 µg/ml DNase I and 14 µM Rho Kinase inhibitor. Cells were split into ~0.5 × 10^6^ cells/well in a 24-well plate in 500 μL of growth media, 500 μL lentivirus, and 8 μg/mL polybrene. Cells were spinfected at 600×*g* for 1 h at room temperature and left to rest at 37 C for 1–6 h. Cells were then replated in a 24-well plate in 35 μL domes/well. Three days later, organoids were dissociated, and transduced cells were sorted by FACS and re-plated at 1000 cells/well in a 96-well plate in 1:1 Matrigel and growth media. 15 min later, 100 μL growth media was added to each well. Organoids were counted 2 weeks later. To image organoids, each well was collected in Cell Recovery Solution and resuspended in growth media in a 96-well U-bottom plate. Images were acquired on a Zeiss Axiovert 40 CFL.

#### Proliferation and cell death analysis

*KP*^*f/f*^*C* or FG cells were infected with shRNA and sorted 72 h later; 50,000 transduced cells were plated in a 24-well plate in 10% DMEM. For BrdU analysis, 24 h after plating, media was refreshed with media containing BrdU (BD Biosciences); after an 18 h pulse in BrdU-containing media, cells were trypsinized, fixed, permeabilized, and stained with anti-BrdU-APC using the BrdU flow cytometry kit (BD Biosciences). For Annexin V analysis, cells were trypsinized and analyzed with the Annexin V apoptosis kit (eBioscience) 48 h after plating.

#### shRNA transplants of KP^f/f^C cells

*KP*^*f/f*^*C* cells were infected with lentiviral RFP-tagged shRNAs against *Smarcd3* or control. 72 h post-transduction, infected stem cells (RFP+Msi2-GFP+ or CD133-APC+) were sorted for transplants. Cells were resuspended at 1000 cells in 50 μL Matrigel plus 50 μL 10% DMEM media; 100 μL tumor cell mixture was injected subcutaneously into the left or right flank of NOD/SCID recipient mice. Flank tumors were measured bi-weekly using calipers for 3 weeks. Tumors were then isolated and dissociated for FACS analysis as described. 24 female NOD/SCID mice between the ages of 6–8 weeks were used for these studies.

#### Secondary syngeneic transplant of KP^f/f^C cells

Mid-point *Smarcd3*^*WT*^*-KP*^*f/f*^*C* and *Smarcd3*^*KO*^*-KP*^*f/f*^*C* tumors (7–8 weeks) were isolated, dissociated and stained for FACS as described. EpCAM-APC+ tumor cells were resuspended at 20,000 cells in 50 μL Matrigel plus 50 μL 10% DMEM media; 100 μL tumor cell mixture was injected subcutaneously into the left flank of immune competent littermate recipients (8 weeks of age). Male and female littermate recipients were used equivalently when possible; littermate recipients did not express Cre. 5 weeks post-transplant flank tumors were isolated, dissociated, and analyzed by FACS as described below.

#### Inducible deletion of Smarcd3 in KPF-R26-CreER^T2^ transplants and mice

Tumors from end-stage *Smarcd3*^*f/f*^*-KPF-R26-CreER*^*T2*^ mice (10–15 weeks of age) were dissociated and sorted as described. EpCAM-APC+ tumor cells were resuspended at 5000 cells in 50 μL Matrigel plus 50 μL 10% DMEM media; 100 μL tumor cell mixture was injected subcutaneously into the left flank of NSG mice (6–8 weeks). Mice were monitored bi-weekly for tumor development. When tumors >3 mm were detected, mice were randomized into IP treatment with tamoxifen (100 mg/kg) or vehicle (100 μL corn oil) for 5 days. Three weeks after enrollment, tumors were isolated and analyzed by FACS as described.

At 6 weeks of age, *KPF-R26-CreER*^*T2*^ mice were enrolled in bi-weekly palpation to track tumor development. Upon tumor detection, Cre+ and Cre- *KPF-R26-CreER*^*T2*^ mice were enrolled in treatment with 150 mg/kg tamoxifen (IP) for 5 days to induce *Smarcd3* deletion. Three weeks after tamoxifen induction, tumors were isolated and dissociated for FACS analysis as described above.

#### Patient-derived xenograft transplants

PDX tumors were dissociated as described. 500,000 tumor cells were plated in 24-well ultra-low attachment plates in 500 μL growth media plus GFP-tagged shRNA (MOI = 25) with 8 μg/mL polybrene. The next day, cells were resuspended in 50 μL organoid media. 15 μL cells were set aside and replated in a 96-well ultra-low attachment plate; these cells were cultured to 48 h post-transduction and stained with EpCAM-PE for FACS analysis to assess transduction efficiency (%GFP+EpCAM-PE+) at *t* = 0. The remaining cells were mixed 1:1 with Matrigel and transplanted into the left flank of NSG recipient mice. 12 weeks later tumors were dissociated for endpoint analysis. PDX shRNA studies in vivo were conducted using three independent samples; one sample was run singly while the other two were run in duplicate across 2 independent shRNA.

#### Tumor initiation studies

Pancreata were isolated from *Smarcd3*^*f/f*^*-Kras*^*G12D/+*^*-Ptf1a-Cre* mice (9–10 weeks of age). Tissue was examined for gross morphology and collected for histological analysis and H&E staining (UCSD Tissue Technology Shared Resource). To induce recombination in ductal or acinar-specific lines, 8-week-old *Smarcd3*^*f/f*^*-Kras*^*G12D/+*^*-Ptf1a*^*CRE-ERTM*^ or *Smarcd3*^*f/f*^*-Kras*^*G12D/+*^*-Sox9-CreER*^*T2*^ mice were treated with 3 doses or 1 dose respectively of 150 mg/kg tamoxifen (in corn oil), IP. 90 days after the first tamoxifen dose, pancreatic tissue was isolated and assessed.

#### Gemcitabine treatment in vivo

At 6 weeks of age, *Smarcd3*^*WT*^*-KP*^*f/f*^*C* and *Smarcd3*^*KO*^*-KP*^*f/f*^*C* mice were weighed and enrolled into treatment with 25 mg/kg gemcitabine in PBS; mice were re-weighed and treated once weekly until humane endpoint for analysis of overall survival (*n* = 6–7 mice per genotype).

#### GC–MS of fatty acids

*Smarcd3*^*f/f*^*-KPF-R26-ER*^*T2*^ flank tumor cell transplants were treated with tamoxifen or vehicle (corn oil); 3 weeks after treatment, tumors were dissociated and ~100,000 EpCAM-APC+ tumor cells were sorted, washed in PBS, and flash frozen for analysis of free fatty acids by gas chromatography–mass spectrometry (GC–MS) at the UCSD Lipidomics Core according to standard protocols. Free fatty acid concentration was normalized to protein concentration.

#### Western blot

Western blot was used to assess protein knockdown in *KP*^*f/f*^*C* and FG cells. shRNA-transduced cells were sorted and plated in 2D culture for 72 h; cells were then lysed in RIPA buffer. Protein was quantified by Bradford assay; 30 μg was denatured at 95 C for 5 min in 4× Laemmli sample buffer and loaded per well in a 4–15% precast Mini-PROTEAN TGX gel. Gels were run at 100 V for 1 h and transferred to PVDF at 90 V/250 mA for 1 h. Blots were blocked in Odyssey buffer incubated in primary antibodies diluted in Odyssey buffer 0.1% Tween20 overnight. Blots were washed and incubated in secondary antibodies (1:10,000, Li-cor) the next day at room temperature for 1 h before images were collected (Li-cor scanner). Antibodies and dilutions are listed in Supplementary Table 2. Uncropped and unscanned images of all blots are included in the Source Data file.

#### IP-Western, co-IP and IP-mass spectrometry

Primary *Smarcd3*^*WT*^ and *Smarcd3*^*KO*^*-KP*^*f/f*^*C* cell lines were derived from end-stage tumors as described above and cells were collected for lysis and analysis of BAF complex composition using immunoprecipitation (IP) followed by western blot or mass spectrometry (MS).

Nuclear lysates were collected following a revised Dignam protocol^69^. After cellular swelling in Buffer A (10 mM HEPES pH 7.9, 1.5 mM MgCl_2_, 10 mM KCl supplemented with 1 mM DTT, 1 mM PMSF, and protease inhibitors) cells were lysed by homogenization using a 21-gauge needle. If lysis remained incomplete, cells were treated with 0.1% Igepal-630 for ten minutes on ice prior to nuclei collection. Nuclei were spun down at 1300×*g* for five minutes then resuspended in Buffer C (20 mM HEPES pH 7.9, 20% glycerol. 420 mM NaCl, 1.5 mM MgCl_2_, 0.2 mM EDTA supplemented with 1 mM DTT, 1 mM PMSF, and protease inhibitors). After 30 min rotation at 4 °C samples were clarified at 21,000×*g* for 10 min. Supernatant was collected, flash frozen and stored in the −80 °C.

For IP-Western, anti-IgG, anti-SMARCA4, anti-BRD9, anti-ARID1A, anti-SMARCD1, and anti-SMARCD3 were used to immunoprecipitate BAF complex subunits from 200 μg of nuclear lysate per IP. Bound proteins from each IP were bound to 50:50 Protein A:Protein G Dynabeads for 1–2 h and washed extensively with IP wash buffer (50 mM Tris pH 8, 150 mM NaCl, 0.2 mM EDTA, 0.1% Igepal-630, 1 mM MgCl_2_). Proteins were eluted in SDS–PAGE loading solution with boiling for 5 min and analyzed by western blot to determine the association of SMARCD3 with the BAF complexes. Antibodies and dilutions are described in Supplementary Table 2.

For IP-MS analysis, anti-IgG or anti-SMARCA4 antibody was used for immunoprecipitation from *Smarcd3*^*WT*^*-KP*^*f/f*^*C* and *Smarcd3*^*KO*^*-KP*^*f/f*^*C* lysates. Antibodies were crosslinked to Protein A:Protein G Dynabeads using bis(sulfosuccinimidyl) suberate (BS3). Dynabeads were blocked with 10 μg/μL sheared salmon-sperm DNA in wash buffer (WB, 0.1 M NaPO_4_ pH 8.2, 0.1% Tween-20) then incubated with antibody at room temperature for 15 min. After two washes with conjugation buffer (20 mM NaPO_4_ pH 8.2, 150 mM NaCl), the antibody-bead complexes were incubated with 5 mM BS3 for 30 min at room temperature. Cross-linking was quenched with Tris–HCl pH 7.4, and complexes were washed with conjugation buffer and equilibrated with IP buffer (50 mM Tris pH 8, 150 mM NaCl, 0.1% Igepal-630). IP was performed as above, but washed with RIPA buffer (50 mM Tris pH 8, 150 mM NaCl, 1% Igepal-630, 0.5% sodium deoxycholate, 0.1% SDS). Proteins were eluted in 20 mM Tris pH 8, 150 mM NaCl, 1x SDS–PAGE loading dye, 10 mM DTT with boiling. Samples were precipitated by methanol/chloroform. Dried pellets were dissolved in 8 M Urea/100 mM TEAB pH 8.5. Proteins were reduced with 5 mM tris(2-carboxyethyl)phosphine hydrochloride and alkylated with 10 mM chloroacetamide. Proteins were digested overnight at 37 °C in 2 M Urea/100 mM TEAB pH 8.5 with trypsin. Digestion was quenched with formic acid, 5% final concentration. The digested samples were analyzed on a Fusion Orbitrap tribrid mass spectrometer (Thermo) in a data-dependent mode.

For co-immunoprecipitation (co-IP) of SMARCD3-FLAG and FOXA1-V5, 293T cells were transduced with lentiviral FOXA1-V and transfected with either SMARCD3-FLAG or empty GFP vector. Cells were collected for nuclear lysis and co-IP using the nuclear co-IP kit by Active Motif. 500 μg of nuclear lysate was incubated with 50 μL FLAG magnetic beads rotating overnight at 4 °C in high stringency buffer. The next day, beads were washed three times in IP High buffer and eluted in 100 μL 1.5 mg/mL FLAG peptide. 4X Laemmli buffer was added to the eluate and boiled at 95 °C for 5 min. 20 μL eluate was used western blot as outline above. Blots were stained with anti-FLAG, anti-V5, or anti-SMARCA4. Antibodies and specific reagents are listed in Supplementary Tables 2 and 3.

#### Immunofluorescence

Pancreatic cancer tissue from *KP*^*f/f*^*C*, *KPC*, *KPF*, *KPF-R26-ER*^*T2*^, *KC*, or PDX tumors was fixed in 10% neutral buffered formalin and paraffin embedded at the UCSD Tissue Technology Shared Resource. 5 µm sections were obtained and deparaffinized in xylene. The human pancreas paraffin embedded tissue array was acquired from US Biomax, Inc. Antigen retrieval was performed for 45 min in 95–100 °C 1x Citrate Buffer. Red blood cells were lysed for 10 min in ammonium chloride. Sections were blocked for 1 h in PBS containing 0.1% Triton X100, 10% Goat Serum, and 5% bovine serum albumin.

Primary *KP*^*f/f*^*C* cells were suspended in DMEM (Gibco, Life Technologies) supplemented with 50% FBS and adhered to slides by centrifugation at 30×*g*. After drying for 15 min, cells were fixed with 4% paraformaldehyde, washed in PBS, and blocked for 1 h with PBS containing 0.1% Triton X-100, 10% Goat serum, and 5% bovine serum albumin. Some cytoplasmic SMARCD3 staining was observed by this cytospin technique but was not verified in any other fixed tissue, which was validated by staining on KO tissue.

Primary antibodies were incubated overnight at 4 °C followed by 45 min incubation with AlexaFluor-conjugated secondary antibodies at room temperature. DAPI was used to detect DNA and images were obtained with a Confocal Leica TCS SP5 II (Leica Microsystems). Signal amplification was used for SMARCD3 staining of mouse or human pancreatic tissue; overnight primary antibody staining was followed by incubation with anti-rabbit biotin antibody for 1 h. Slides were then incubated with AlexaFluor streptavidin 568, DAPI, and Alexa-Fluor-conjugated secondary antibodies for 45 min at room temperature. Antibodies are described in Supplementary Table 2.

For proximity ligation assays, tissue processing was performed as described above and the proximity ligation assay was performed per the manufacturer’s protocol (DuoLink PLA detection); amplification was 2 hours. DuoLink rabbit probes (MINUS) and goat probes (PLUS) were used. Images were analyzed using ImageJ software version 1.50i. Antibodies, reagents, and software are described in Supplementary Tables 2 and 3.

#### Immunohistochemistry

Total area of H&E-stained tumor sections was analyzed using QuPath. Tumors were isolated and cut in half along their longest diameter; tissue was fixed in 10% neutral buffered formalin and paraffin-embedded, sectioned, H&E-stained, and scanned at the UCSD Tissue Technology Shared Resource. H&E sections cut from the largest middle plane was used for QuPath analysis of the tumor area. Thresholding was used to detect whole tissue and live H&E-stained tumor tissue; parameters were saved as a classifier and applied to each section for tissue and live tumor tissue detection as well as tumor area measurements. To analyze tumor cell number, serial sections were stained with hematoxylin to identify nuclei and used to train an object classifier in QuPath to detect tumor and stromal cells and regions of necrosis. This object classifier was applied to all stained sections and used to detect and count the total tumor cell number within the entire tissue slice region.

#### Pathological analysis of mouse tumors

Pancreatic tissue from KC, *Kras*^*LSL/+*^; *Ptf1a-Cre*^*ER*^ (acinar), and *Kras*^*LSL/+*^; *Sox9-Cre*^*ER*^ mice were isolated as described above. H&E slides were scanned and multiple fields of view were randomly acquired; all ductal or PanIN-like structures were annotated in each image and sent to a pathologist (V.V.) for blinded analysis. V.V. annotated the PanIN grade of all lesions in all images.

#### Analysis of clinically annotated TMA

TMAs were sectioned to 2.5 µm thickness. IHC staining was performed on a Leica BOND RX automated immunostainer using BOND primary antibody diluent and BOND Polymer Refine DAB Detection kit per manufacturer’s instructions (Leica Biosystems). Pre-treatment was performed using citrate buffer at 100 °C for 30 min, and tissue was stained using rabbit anti-human Smarcd3 antibody (Aviva Systems Biology). Slides were scanned using a Pannoramic P250 digital slide scanner (3DHistech). Smarcd3 staining of TMA spots was analyzed in an independent and randomized manner by two board-certified surgical pathologists (C.M.S and M.W.) using Scorenado, a custom-made online digital TMA analysis tool. Interpretation of staining results was in accordance with the “reporting recommendations for tumor marker prognostic studies” (REMARK) guidelines. Equivocal and discordant cases were reviewed by a third board-certified surgical pathologist (E.K.) to reach a consensus. Smarcd3 staining in tumor cells was classified microscopically as negative (absence of any staining), vs. positive (any positive staining in tumor cells). Spots/patients with no interpretable tissue (less than 10 intact, unequivocally identifiable tumor cells) or other artifacts were excluded.

#### RT-qPCR

RNA was isolated using RNeasy Micro and Mini kits (Qiagen) and converted to cDNA using Superscript III (Invitrogen). Quantitative real-time PCR was performed using an iCycler (BioRad) by mixing cDNAs, iQ SYBR Green Supermix (BioRad), and gene-specific primers. All real-time data was normalized to B2M. Primer sequences are available in Supplementary Table 4.

#### Viral constructs and production

Short hairpin RNA (shRNA) constructs against mouse genes were designed using the Broad RNAi consortium and cloned into the lentiviral pLV-hU6-mPGK-red vector by Biosettia. shRNA against human genes were designed using the Broad RNAi consortium and cloned into the lentiviral FG12 vector. Single guide RNA (sgRNA) constructs were designed using Benchling and cloned into the GeCKO lentiv2 vector; lentiCRISPR v2 was a gift from Feng Zhang (Addgene plasmid #52961). Oligonucleotide sequences are described in Supplementary Table 4. GFP-tagged lentiviral human *SMARCD3* overexpression vector and IRES-GFP control were provided by Dr. Pier Lorenzo Puri. FOXA1-V5 plasmid pLX302_FOXA1-V5 was a gift from William Hahn (Addgene plasmid # 70090; http://n2t.net/addgene:70090; RRID:Addgene_70090). Inducible shKras plasmid TGMP.shKras.1422 was a gift from Tyler Jacks (Addgene plasmid # 59913; http://n2t.net/addgene:59913; RRID:Addgene_59913). Virus was produced in 293T cells transfected with 4 µg shRNA constructs along with 2 µg pRSV/REV, 2 µg pMDLg/pRRE, and 2 µg pHCMVG constructs. Viral supernatants were collected for two days then concentrated by ultracentrifugation at 50,000×*g* for 2 h at 4 °C. Adenoviral GFP and Cre were purchased from the viral vector core at the University of Iowa. Plasmids are listed in Supplementary Table 3.

#### In vitro pulse chase experiment

To assess the stability of SMARCD3, 350,000 *KPF* cells/well were plated in a six-well plate. At >75% confluency, the media was replaced with DMEM without glucose, 1% pen/strep, 1% NEAA, and 1 mM or 10 mM glucose. The cells were treated with 100 pg/ml or 10 ng/ml actinomycin for 4 or 8 h to assess mRNA stability or with 1 μg/ml cycloheximide for 8, 12, or 24 h to assess protein stability. Subsequently, actinomycin-treated samples were subjected to RNA isolation and cycloheximide-treated samples were collect and lysed for western blotting.

### Genome-wide sequencing

#### Analysis of SMARCD3+ cells within human PDAC scRNA-seq

Human PDAC single-cell RNA sequencing^40^ was aligned to the 10X Genomics pre-built hg38 reference, and feature-barcode matrices were generated using Cell Ranger v3. Secondary analysis was performed using the Seurat v3.1R package. Cells were filtered for a minimum of 500 features, a maximum of 2500 features, and a mitochondrial percentage <10% per cell. Read counts were normalized using log normalization and 2000 variable features were identified using a vst selection method. PCA dimensionality reduction was performed, and elbow plots were used to determine dimensionality. Cluster resolutions were adjusted between 0.3 and 0.6 accordingly to obtain discrete gene signatures among the clusters. Uniform Manifold Approximation (UMAP) was used to render final single-cell composition plots. Cells were gated on *EPCAM*+ and *SMARCD3*+ cells were quantified within *EPCAM*+ cells, *EpCAM*+*PROM1*+(CD133+) cells, and *EPCAM*+*MSI2*+ cells. Software and algorithms are published and are listed in Supplementary Table 3.

#### RNA-sequencing

Low-passage primary CD133^High^ *KP*^*f/f*^*C* tumor cells were derived as outlined above. 1 × 10^6^ cells were infected with RFP-tagged shRNA against *Smarcd3* or control in triplicate; transduced cells were sorted 72 h post-transduction and plated in a 10 cm plate in 10% DMEM growth media. 5 days later, cells were collected for analysis by RNA-seq and ChIP-seq. >300,00 cells per replicate were collected for RNA-seq; total RNA was isolated using Quick-RNA Miniprep Kit (Zymo Research). RNA was assessed for quality using an Agilent Tapestation; all samples had RIN > 7. Libraries were generated from 100 ng RNA using Illumina’s TruSeq Stranded mRNA Sample Prep Kit following the manufacturer’s instructions.

#### ChIP-sequencing

*KP*^*f/f*^*C* cells were transduced and plated as above for RNA-seq and ChIP-seq analysis. For SWI/SNF ChIP-seq, 6−7e^6^ cells were collected and cross-linked in 3 mM disuccinimidyl glutarate and then in 1% formaldehyde. For histone modification ChIP-seq, 2e^6^ cells were collected and cross-linked with 1% formaldehyde. After quenching excess formaldehyde with 125 mM glycine, fixed cells were washed, pelleted and flash-frozen. Upon thawing, cells were resuspended in lysis solution (50 mM HEPES–KOH pH 8, 140 mM NaCl, 1 mM EDTA, 10% glycerol, 0.5% NP40, 0.25% Triton X-100) and incubated on ice for 10 min. The nuclei were washed with wash solution (10 mM Tris–HCl pH 8, 1 mM EDTA, 0.5 mM EGTA, 200 mM NaCl) and shearing buffer (0.1% SDS, 1 mM EDTA, 10 mM Tris–HCl pH 8) then sheared in a Covaris E229 sonicator for 10 min to generate DNA fragments between ~200 and 1000 bp. After clarification by centrifugation, chromatin was immunoprecipitated overnight at 4 °C with antibodies against SMARCA4, ARID1A, FOXA1, KLF5, H3K4me, H3K4me3, and H3K27ac (antibodies listed in Supplementary Table 2), then bound to Protein A+G Dynabeads in ChIP buffer (50 mM HEPES–KOH pH 7.5, 300 mM NaCl, 1 mM EDTA, 1% Triton X-100, 0.1% DOC, 0.1% SDS). Antibody-bound DNA were washed and treated with Proteinase K and RNase A and the purified ChIP DNA was used for library generation (NuGen Ovation Ultralow Library System V2) for sequencing.

#### Analysis of RNA-seq and ChIP-seq

Reads were aligned to the mouse genome (mm10) using STAR (v2.5) for RNA-seq and ChIP-seq. Only reads that mapped to a unique genomic location (MAPQ > 10) were used for downstream analysis. HOMER (v4.8, http://homer.salk.edu/homer/) was used to process alignment files to generate ChIP-seq bed files. ChIP-seq peaks for SMARCA4 and ARID1A were found by using the findPeaks program in HOMER with the parameter “-style factor” versus the appropriate ChIP input experiments as background. ChIP-seq peaks for H3K4me, H3K4me3, and H3K27ac were called using the parameter “-style histone”. SMARCA4 and ARID1A peaks were called when enriched >four-fold over input and local tag counts (FDR 0.001, Benjamin–Hochberg). For histone ChIP-seq, peaks within a 1000 bp range were stitched together to form regions. Differential ChIP-seq peaks were found by merging peaks from shControl and shSmarcd3 groups and called using getDifferentialPeaks with fold change 1.5, Poisson *p* value < 0.0001. For motif enrichment analysis, sequences within 200 bp of peak centers were compared to motifs in the HOMER database using the findMotifsGenome.pl command using default fragment size and motif length parameters. Random GC content-matched genomic regions were used as background. Enriched motifs are statistically significant motifs in input over background by a *p* < 0.05 using cumulative binomial distribution.

For RNA-seq, RNA expression was quantified as raw integer counts using analyzeRepeats.pl in HOMER using the following parameters: -strand both -count exons -condenseGenes -noadj. To identify differentially expressed genes, we performed getDiffExpression.pl in HOMER, which uses the DESeq2 R package to calculate the biological variation within replicates. Cut-offs were set at log2 FC = 0.585 and FDR at 0.05.

Software and algorithms are published and are listed in Supplementary Table 3.

#### GSEA

Gene set enrichment analysis (GSEA) was performed with the Bioconductor GSVA data C2, C6, and C7 BroadSets gene set collections from MsigDB3.0. Additionally, we used curated gene sets we derived from published data in context of shFoxa1 or sgFoxa1 knockdown^49^, sgKlf5 knockdown^70^, and a gene signature enriched within *Msi2*+*KP*^*f/f*^*C* stem cells and *Msi2-KP*^*f/f*^*C* non-stem cells^8^. Software and algorithms are published and are listed in Supplementary Table 3.

#### Network analysis

Genes down-regulated by Smarcd3 knockdown (padj < 0.05, logFC < −0.35) were used to construct a network using high confidence (>0.8) interactions within the STRING mouse interactome in Cytoscape. The STRING network contained 1030 nodes connected by 7860 edges; node size was scaled to logFC by RNA-seq. To interrogate functional programs, we applied a community clustering algorithm (GLay) to the network using clusterMaker. This generated 12 network hubs of interacting proteins; we used STRING functional enrichment to identify enriched annotations for each hub. We pulled all genes from four lipid-implicated hubs into a “lipid subnetwork”. We labeled specific lipid-associated nodes and overlaid ChIP-seq data on this network to identify nodes bound by SMARCD3-BAF and FOXA1. Node genes that were co-bound by FOXA1 and lost SMARCA4/ARID1A binding by ChIP-seq were considered putative targets of SMARCD3-BAF/FOXA1; direct targets with lipid functions were highlighted with a yellow node label, yellow diamond-shaped node, and manually inserted yellow edges indicating regulation by SMARCD3-BAF/FOXA1. Software and algorithms are published and are listed in Supplementary Table 3.

### Quantification and statistical analysis

Statistical analyses were carried out using GraphPad Prism software version 8.2.0 (GraphPad Software Inc.). Sample sizes for in vivo studies were determined based on the variability of pancreatic tumor models used. For flank transplant and autochthonous drug and tamoxifen studies, tumor-bearing animals within each group were randomly assigned to treatment groups. Experimental group sizes were determined based on previous studies^7,8^. Data are shown as the mean ± SEM. Two-tailed unpaired Student’s *t*-tests with Welch’s correction or 1-way or 2-way analysis of variance (ANOVA) for multiple comparisons when appropriate were used to determine statistical significance; *p* values were adjusted for multiple comparisons in the case of analysis by ANOVA.

The level of replication for each in vitro and in vivo study is noted in the figure legends for each figure and described in detail above. To summarize, in vitro sphere or colony formation studies were conducted with *n* = 3 independent wells per cell line across two independent shRNA of *n* = 3 wells; the majority of these experiments were additionally completed in >2 independently derived cell lines, *n* = 3 wells per shRNA. Because material was limited, PDX organoids treated with shRNA were plated in *n* = 3–4 wells per experiment, for one experiment each using two independent PDX organoid lines. Flank shRNA studies were conducted three times using independent cell lines, with *n* = 3–4 tumors per group in each experiment. Analysis of midpoint (7–8 weeks old) *KP*^*f/f*^*C* tumors was conducted with *n* = 5–16 mice per group. Secondary syngeneic transplants were conducted with *n* = 3–4 independent tumors per group, transplanted into *n* = 2–4 littermate recipients each. Survival studies in *KP*^*f/f*^*C* mice plus and minus gemcitabine treatment were conducted with *n* = 6–10 mice per group. Flank *KPF*+adCre and *KPF-R26-CreER*^*T2*^ tamoxifen treated transplants were conducted in 2 biological replicates at *n* = 3–5 tumors per group. Tumor initiation studies in the autochthonous *KC* model were conducted with *n* = 3–9 mice for all Cre systems used. Three independent PDX tumors were used for shRNA studies in vivo, one PDX sample was used for one experiment while the other two were completed in duplicate for a total of *n* = 4–5 per shRNA for 2 independent shRNA. Source data for all studies is available in the Source Data file and specific details regarding animal studies are available in Supplementary Data 2. RNA-seq in *KP*^*f/f*^*C* cells was run in triplicate, H3K27-acetyl ChIP-seq was run in duplicate, and one ChIP each was run for H3K4me, H3K4me3, SMARCA4, and ARID1A ChIP-seq.

### Reporting summary

Further information on research design is available in the Nature Portfolio Reporting Summary linked to this article.

## Supplementary information

Supplementary Information

Description of Additional Supplementary Files

Supplementary Data 1

Supplementary Data 2

Reporting Summary

## Data Availability

The *KP*^*f/f*^*C* RNA-seq and H3K27-acetyl, H3K4me, H3K4me3, ARID1A, KLF5, FOXA1 and SMARCA4 ChIP-seq data generated in this study have been deposited in the GEO database under accession code GSE168490. The processed RNA-seq data are available also available under accession code GSE168490. The IP-MS data generated in this study have been deposited in MassIVE under accession MSV000090744 (https://massive.ucsd.edu/ProteoSAFe/dataset.jsp?task=63becd806f68431c90cb6455e5e7ec12) and in the ProteomeXchange dataset under the accession number PXD038216. The publicly available mouse Msi2+ and Msi2− primary *KP*^*f/f*^*C* RNA-seq data^8^ used in this study are available in the GEO database under accession code GSE114906. The publicly available human PDAC scRNA-seq data^40^ used in this study are available at the Genome Sequence Archive under accession code GSA: CRA001160. Source data are provided in this paper. The remaining data are available within the Article, Supplementary Information, or Source Data file. Source data are provided in this paper.

## Source data

Source Data

## Code availability

The digital TMA analysis tool, Scorenado is available on github: https://github.com/digitalpathologybern/scorenado.
