# Supplementary material for: Smarcd3 is an epigenetic modulator of the metabolic landscape in pancreatic ductal adenocarcinoma
Source: Nat Commun. 2023 Jan 18;14:292. doi: 10.1038/s41467-023-35796-7 (PMC9849267; doi:10.1038/s41467-023-35796-7)
Supplement: Supplementary file 1 — Supplementary Information [file 41467_2023_35796_MOESM1_ESM.pdf]

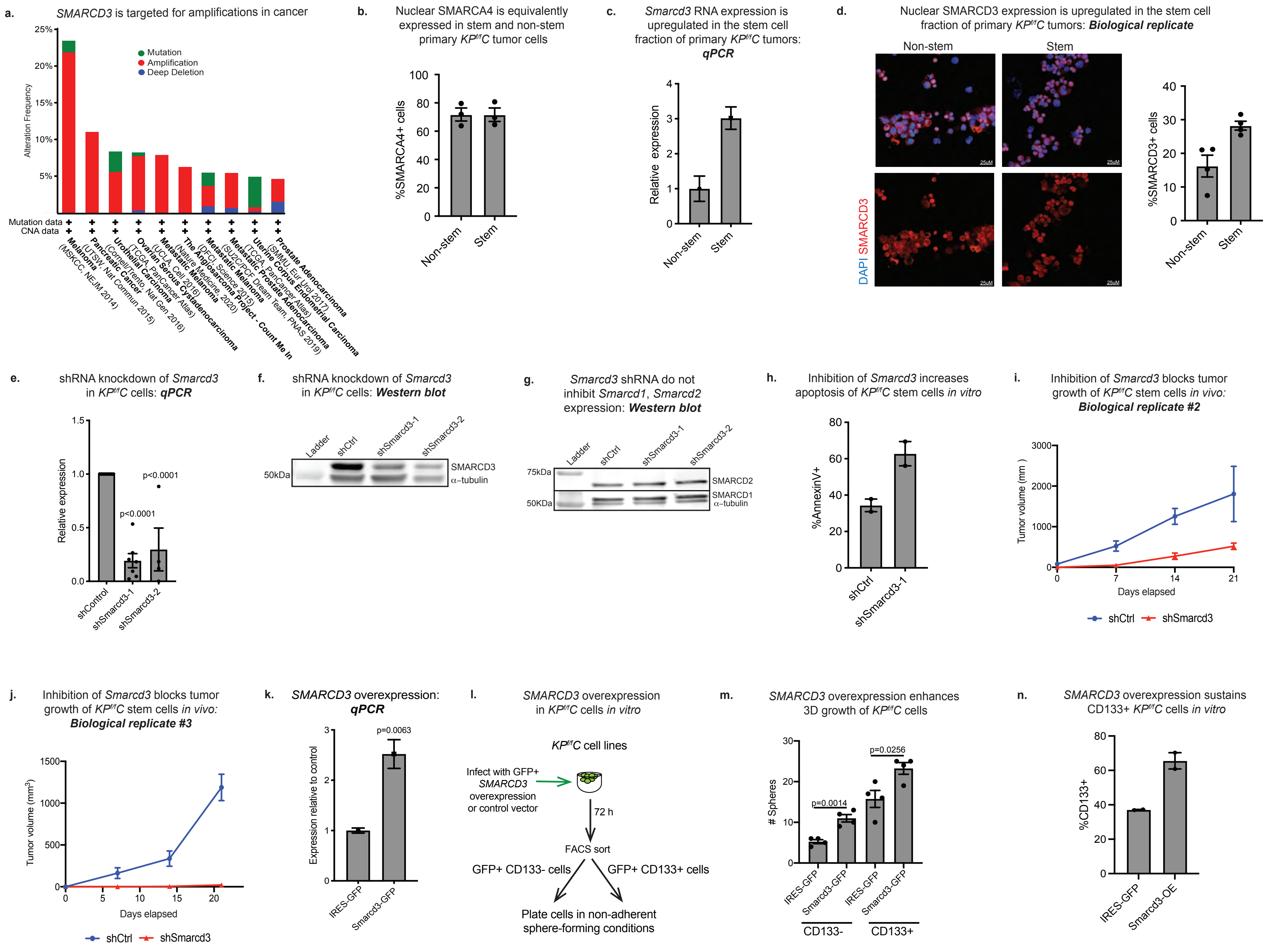

SUPPLEMENTARY FIGURE 1

Supplementary Figure 1: SMARCD3 is a functional epigenetic dependency in PDAC

a. *SMARCD3* is targeted for amplifications in clinical cancer cases (top 10 studies with the highest frequency of *SMARCD3* alteration, cBioPortal; see Figure 1d).

b. SMARCA4 is equivalently expressed in stem and non-stem *KP*$^{f/f}$*C* tumor cells. Stem (CD133+) and non-stem (CD133-) EpCAM+ tumor cells were sorted from primary *KP*$^{f/f}$*C* tumors and analyzed for nuclear SMARCA4 expression by immunofluorescence (n=3 frames, n=1 biological replicate, mean ± SEM).

c. *Smarcd3* expression is upregulated within the stem cell fraction of a primary *KP*$^{f/f}$*C* tumor. Stem (CD133+) and non-stem (CD133-) *KP*$^{f/f}$*C* tumor cells were sorted; *Smarcd3* expression assessed by qPCR (n=1 biological replicate at n=2, mean ± SEM).

d. Nuclear SMARCD3 expression is upregulated the stem cell fraction of primary *KP*$^{f/f}$*C* tumors. DAPI (blue), SMARCD3 (red); tumor cells with positive staining for SMARCD3 in the nucleus were counted (n=2 biological replicates, n=3 frames; mean ± SEM). See Figure 1f.

e. *Smarcd3* knockdown in *KP*$^{f/f}$*C* cells *in vitro:* qPCR (biological replicates are n=7 for shSmarcd3-1 and n=4 for shSmarcd3-4 at n=3 each; 2-way ANOVA with multiple comparisons, mean ± SEM).

f. *Smarcd3* knockdown in *KP*$^{f/f}$*C* cells: Western blot (representative of n=2).

g. *Smarcd3* shRNA are specific and do not reduce SMARCD1 or SMARCD2 expression in *KP*$^{f/f}$*C* cells: western blot (representative of n=2).

h. *Smarcd3* knockdown increases apoptosis (Annexin V positivity) of CD133+ *KP*$^{f/f}$*C* cells *in vitro* (n=2, mean ± SEM).

i. *Smarcd3* knockdown blocks growth of *KP*$^{f/f}$*C* stem cells *in vivo.* Biological replicate #2. *Smarcd3* knockdown blocks growth of Msi2+ *KP*$^{f/f}$*C* flank transplants (n=2 shCtrl, n=4 shSmarcd3, mean ± SEM; n=3 biological replicates, see Supplementary Figure 1j, Figure 1j).

j. *Smarcd3* knockdown blocks growth of *KP*$^{f/f}$*C* stem cells *in vivo.* Biological replicate #3. *Smarcd3* knockdown blocks growth of Msi2+ *KP*$^{f/f}$*C* flank transplants (n=4 shCtrl, n=2 shSmarcd3, mean ± SEM; n=3 biological replicates, see Supplementary Figure 1i, Figure 1j).

k. *SMARCD3* overexpression in *KP*$^{f/f}$*C* cells *in vitro*: qPCR *KP*$^{f/f}$*C* cells were transduced with lentiviral GFP-tagged *SMARCD3* overexpression vector or empty GFP control and collected for qPCR analysis; one biological replicate (n=3, mean ± SEM).

l. *SMARCD3* overexpression in *KP*$^{f/f}$*C* cells *in vitro*.

m. *SMARCD3* overexpression enhances 3D growth of CD133- and CD133+ *KP*$^{f/f}$*C* cells *in vitro. KP*$^{f/f}$*C* cells transduced with *SMARCD3-GFP* or empty GFP vectors were plated in sphere-forming assay (representative of 4 biological replicates; n=4, two-tailed T-tests, mean ± SEM).

n. *SMARCD3* overexpression sustains CD133+ *KP*$^{f/f}$*C* cells *in vitro. KP*$^{f/f}$*C* cells transduced with *SMARCD3-GFP* or empty GFP vectors were sorted on CD133+ and plated in 2D. %CD133+ positivity was assessed by FACS after 72 h (2 biological replicates; n=2 each, mean ± SEM).

All source data are provided in the Source Data file.

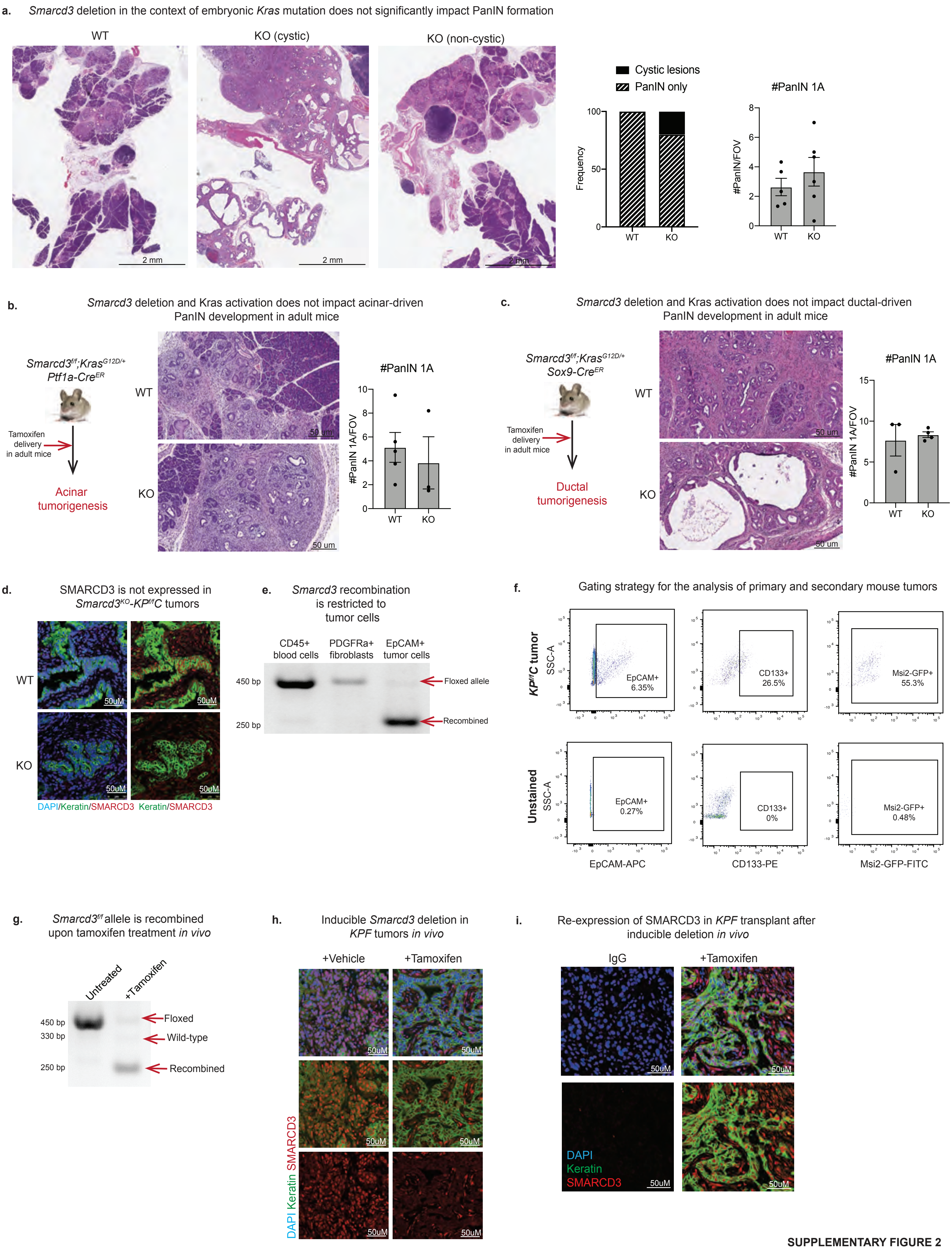

SUPPLEMENTARY FIGURE 2

Supplementary Figure 2: Genetic deletion of Smarcd3 impairs tumor growth

a. *Smarcd3* deletion in the context of embryonic *Kras* mutation does not impact PanIN formation. *Smarcd3^WT^* (WT) and *Smarcd3^KO^* (KO) *Kras^G12D/+^; Ptf1a-Cre* (*KC*) mice (9-10 weeks) were analyzed for morphology and PanIN grade/number (n=8 WT and n=10 KO mice for histological analysis; n=5 WT and n=6 KO mice for analysis of PanIN 1A, representative of n=3 fields of view, mean ± SEM, scale bar = 2mm).

b. *Smarcd3* deletion with *Kras* mutation does not impact acinar-driven PanIN development in adult mice. *Smarcd3^WT^* (WT) and *Smarcd3^KO^* (KO) *Kras^G12D/+^; Ptf1a-CreER^T2^* mice (8 weeks of age) were treated with tamoxifen to induce *Smarcd3* deletion and *Kras* mutation in adult acinar cells. 90 days post-induction, pancreatic tissue was isolated for analysis of PanIN grade/number (representative of n=5 fields of view, n=5 WT and n=3 KO mice, mean ± SEM, scale bar = 50um).

c. *Smarcd3* deletion with *Kras* mutation does not impact ductal-driven PanIN development in adult mice. *Smarcd3^WT^* (WT) and *Smarcd3^KO^* (KO) *Kras^G12D/+^; Sox9-CreER^T2^* mice (8 weeks of age) were treated with one dose of 150mg/kg tamoxifen to induce *Smarcd3* deletion and *Kras* mutation in adult pancreatic ductal cells. 90 days post-induction, pancreatic tissue was isolated for analysis of PanIN grade/number (representative of n=5 fields of view for n=3 WT and n=4 KO mice per genotype, mean ± SEM, scale bar = 50um).

d. SMARCD3 is not expressed in *Smarcd3^KO^-KP^f/f^C* tumors. SMARCD3 (red) staining in tumor cells (pan-keratin+, green) of end-stage *Smarcd3^WT^-KP^f/f^C* (WT) and *Smarcd3^KO^-KP^f/f^C* (KO) tumors; DAPI (blue) (representative, n=3 mice, scale bar = 50um).

e. *Smarcd3* recombination is restricted to tumor cells in *Smarcd3^f/f^-KP^f/f^C* mice. To confirm that *Smarcd3* recombination was restricted to pancreatic tumor cells, *Smarcd3^f/f^-KP^f/f^C* tumors were stained and sorted for CD45+ blood cells, PDGFRa+ fibroblasts and EpCAM+ tumor cells; PCR was used to detect *Smarcd3* recombination which was restricted to EpCAM+ tumor cells (representative, n=2).

f. Gating strategy for all primary and secondary mouse tumors. Representative FACS plots demonstrate the gating strategy used for the analysis of tumor (EpCAM-APC+) and CD133+ (CD133-PE+) and Msi2+ (Msi2-GFP+) tumor stem cells in primary (Figure 2a,b) and secondary (Figure 2c,d) *Smarcd3^WT^* and *Smarcd3^KO^-KP^f/f^C* tumors. FACS plots for unstained tumor cells are shown as a control. Plots are shown for populations that were first gated through morphology (FSC-A/SSC-A), single cell (FSC-A/FSC-H) and live cell (Propidium iodide negative) gates. This gating strategy was applied to the analysis of tumors from all following genetically engineered mouse models and transplants.

g. *Smarcd3^f/f^* allele is recombined upon tamoxifen treatment *in vivo*. EpCAM+ tumor cells were isolated from tamoxifen-treated *Smarcd3^f/f^-KPF-R26-CreER^T2^* flank transplants for PCR (representative, n=6).

h. Tamoxifen delivery drives *Smarcd3* deletion in the *KPF* model *in vivo.* SMARCD3 expression (red) in tumor cells (pan-keratin+, green) of *Smarcd3^f/f^-KPF-R26-CreER^T2^* flank transplants treated with tamoxifen or vehicle; DAPI (blue) (representative, n=1 biological replicate, n=6 transplants stained, scale bar = 50um).

i. SMARCD3 is re-expressed in *KPF* transplant after inducible deletion *in vivo.* SMARCD3 expression (red) in tumor cells (pan-keratin+, green) of *Smarcd3^f/f^-KPF-R26-CreER^T2^* flank transplant treated with tamoxifen; DAPI (blue) (representative, n=1 biological replicate, n=6 transplants stained; one of three tamoxifen-treated transplants re-expressed SMARCD3, scale bar = 50um).

All source data are provided as a Source Data file.

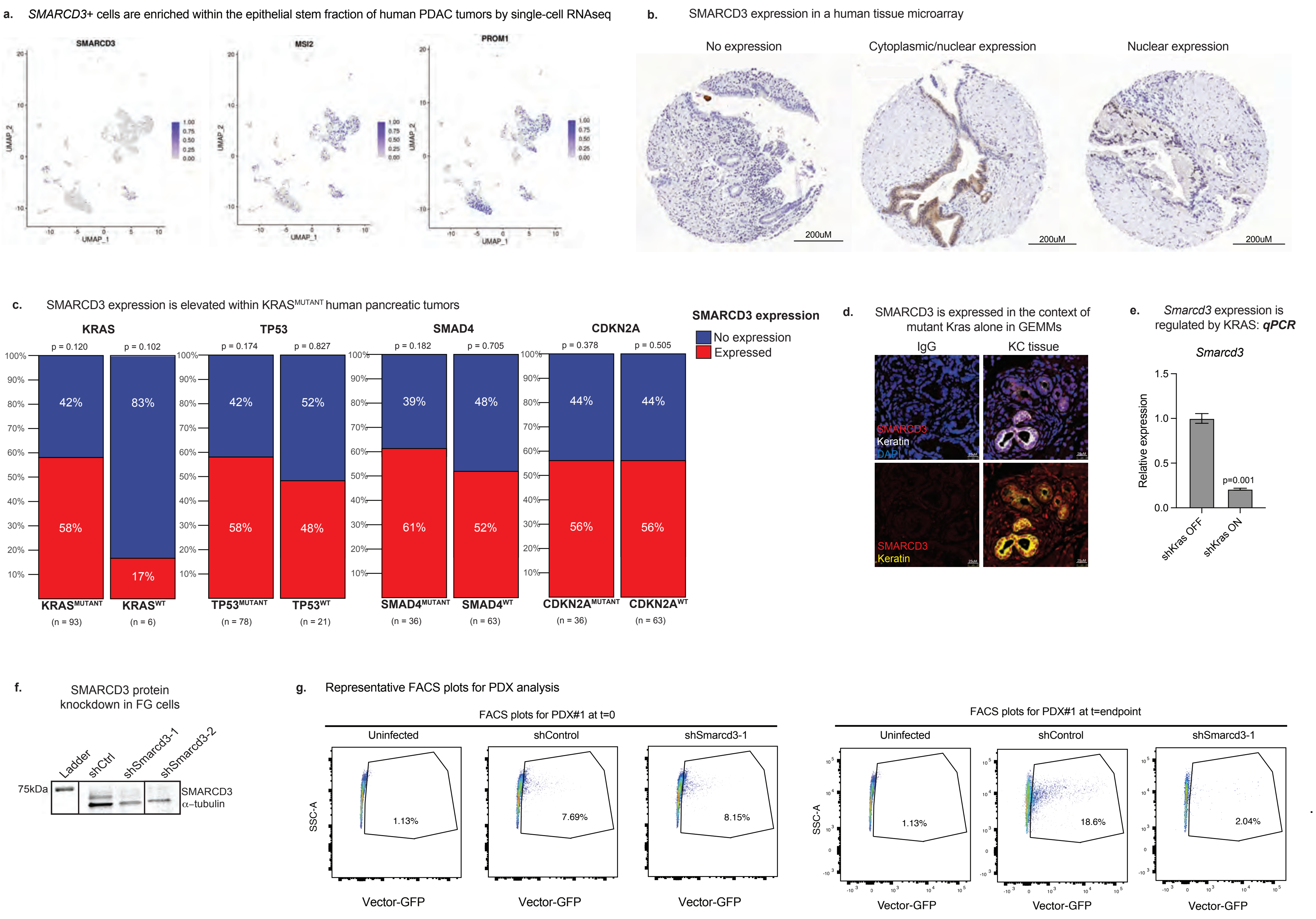

SUPPLEMENTARY FIGURE 3

Supplementary Figure 3: SMARCD3 knockdown blocks tumor growth in human models of PDAC

a. The frequency of *SMARCD3*+ cells is increased in the stem fraction of primary human PDAC tumors by single-cell RNA-seq. After gating on EpCAM+ tumor cells, plots are shown for *SMARCD3*, *PROM1* (CD133+), and *MSI2* expressing cells by single-cell RNA-seq (see Figure 3b).

b. SMARCD3 expression in a human tissue microarray. SMARCD3 expression was assessed by IHC in a cohort of PDAC patients; samples were scored as negative (no expression, left) or positive (any SMARCD3 expression in the nuclei/cytoplasm, middle; or nuclei alone, right) (representative, n=116 cases at n=10 spots/case, scale bar = 200um).

c. SMARCD3 expression is elevated within $KRAS^{MUTANT}$ human pancreatic tumors. SMARCD3 expression was analyzed by IHC in a human tissue microarray where patient tumors were also sequenced (Supplementary Figure 3b). Although non-significant (chi squared test), SMARCD3 expression was most associated with *KRAS* mutation; 58% of $KRAS^{MUTANT}$ tumors expressed SMARCD3 compared to only 17% of $KRAS^{WT}$ tumors.

d. SMARCD3 is expressed in the context of mutant *Kras* alone in GEMMs. SMARCD3 (red) was expressed in PanINs (pan-keratin+, white/yellow) in $Kras^{G12D/+}$*; Ptf1a-Cre (KC)* mice; DAPI (blue) (representative, n=3 mice, scale bar = 25um).

e. *Smarcd3* expression is regulated by *Kras.* $KP^{f/f}C$ tumor cells were transduced with a doxycycline-inducible and GFP-tagged *Kras* shRNA; GFP- (shKras off) and GFP+ (shKras on) cells were sorted for qPCR (n=3 for n=3 biological replicates, two-tailed T-test, mean ± SEM).

f. *SMARCD3* knockdown in FG cells. Human FG PDAC cells transduced with *Smarcd3* shRNA were collected for western blot analysis (α-tubulin loading control, representative, n=2).

g. Representative FACS plots for patient-derived xenograft (PDX) tumor analysis. The frequency of transduced GFP+EpCAM+ PDX tumor cells was analyzed by FACS 48 h post-transduction (t=0). At endpoint (12 weeks), xenograft tumors were dissociated, and analyzed by FACS. Frequency of GFP+ tumor cells are plotted and are gated through live, single EpCAM-PE+ cells (see also Figure 3k).

All source data are provided as a Source Data file.

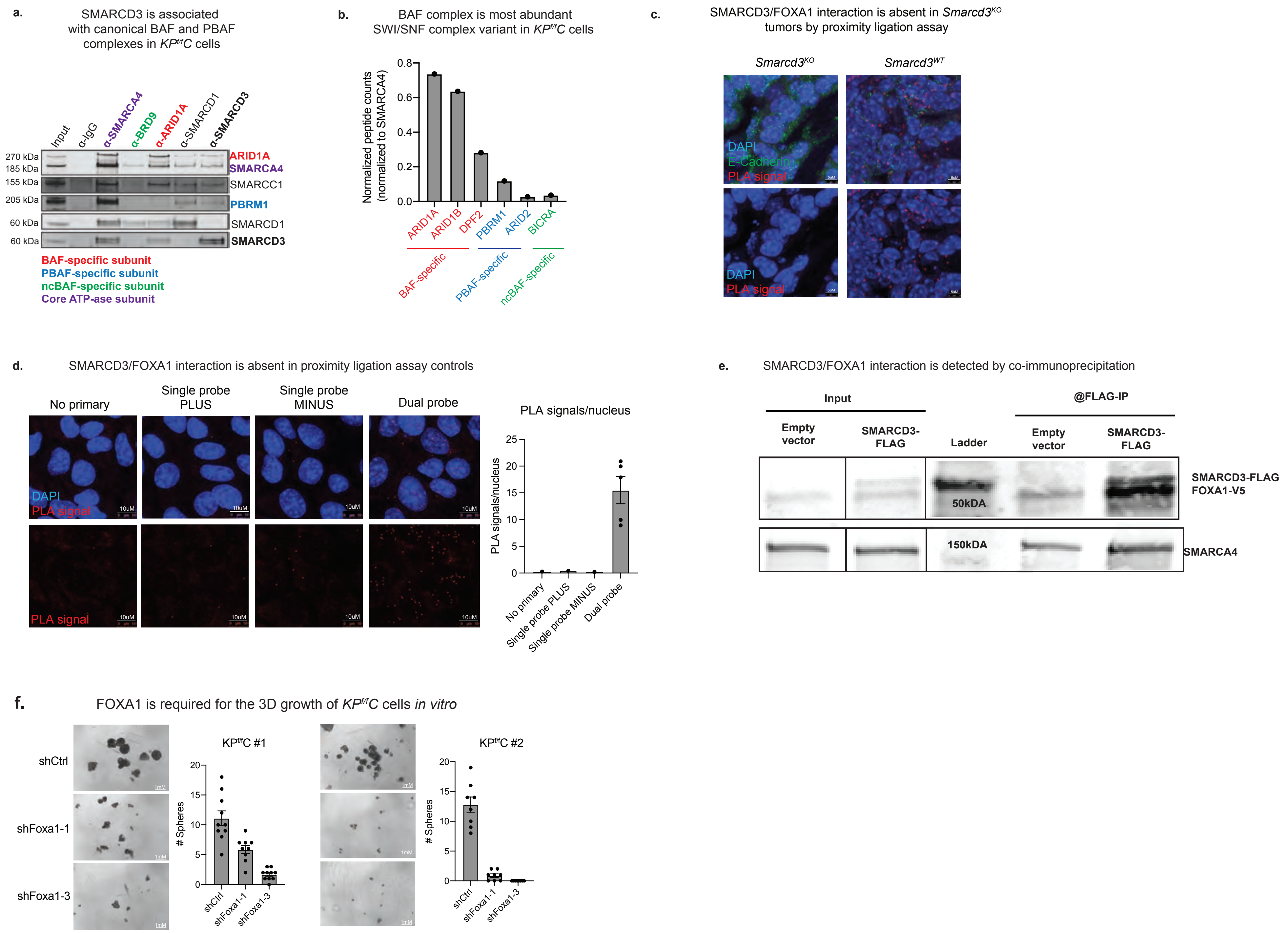

SUPPLEMENTARY FIGURE 4

Supplementary Figure 4: SMARCD3 regulates the epigenetic landscape and BAF complex binding at FOXA1 binding sites in mouse pancreatic cancer cells

a. SMARCD3/Baf60c is associated with canonical BAF and PBAF complexes in *KP*$^{f/f}$*C* cells. Immunoprecipitation (IP) followed by western blot using antibodies against variant-specific SWI/SNF (BAF) complex subunits showed SMARCD3 is associated with BAF (ARID1A) and PBAF (PBRM1) as well as the core ATP-ase subunit SMARCA4 in *KP*$^{f/f}$*C* cells. SMARCD3 does not associate with ncBAF (BRD9) (n=1).

b. BAF is the most abundant SWI/SNF complex variant in *KP*$^{f/f}$*C* cells. *KP*$^{f/f}$*C* cells were collected and SMARCA4 was immunoprecipitated (IP) from the lysates; lysate from the IP was used for mass spectrometry (MS) analysis of SMARCA4-associated proteins. Counts were normalized to bait (SMARCA4); canonical BAF complex members ARID1A, ARID1B, and DPF2 were more abundant than PBAF members PBRM1/ARID2 or ncBAF member BICRA.

c. SMARCD3/FOXA1 interaction is absent in *Smarcd3*^{KO} tumors by proximity ligation assay (PLA). Using antibodies against FOXA1 and SMARCD3, PLA signal (red) was absent in *Smarcd3*^{KO}*-KPF* tumor cells (E-Cadherin, green), serving as a control for the specificity of the PLA reaction; DAPI (blue) (representative from n=2 mice, n=5 frames/tumor, scale bar = 5um; see Figure 4e).

d. SMARCD3/FOXA1 interaction is absent in proximity ligation assay controls. We ran proximity ligation for SMARCD3/FOXA1 using cells stained with no primary antibody and dual probes (left), dual antibodies and single probes (middle), and dual antibodies and probes (complete reaction, right). Although non-specific PLA signal (red) can be detected, it is rarely localized in the nuclei (DAPI, blue). PLA signal was <0.5/nuclei in all controls while nuclear PLA signal was >15/nuclei in the complete reaction in *KP*$^{f/f}$*C* cells (n=1 biological replicate at n=1 frame for probe controls, n=1 biological replicate for dual probe reaction at n=5 frames, scale bar = 10um, mean ± SEM).

e. SMARCD3/FOXA1 interaction is detected by co-immunoprecipitation. SMARCD3-FLAG or empty GFP vector were over-expressed in 293T cells stably co-expressing FOXA1-V5. Cells were collected for co-immunoprecipitation with anti-FLAG. FOXA1-V5 and SMARCA4 co-immunoprecipitated with SMARCD3-FLAG (representative, n=2).

f. FOXA1 is required for the 3D growth of *KP*$^{f/f}$*C* cells *in vitro. KP*$^{f/f}$*C* cell lines were transduced with *Foxa1* shRNA and plated in sphere-forming conditions (representative images from n=2 biological replicates, n=8 and n=10 wells each, mean ± SEM, scale bar = 1mm).

All source data are provided as a Source Data file.

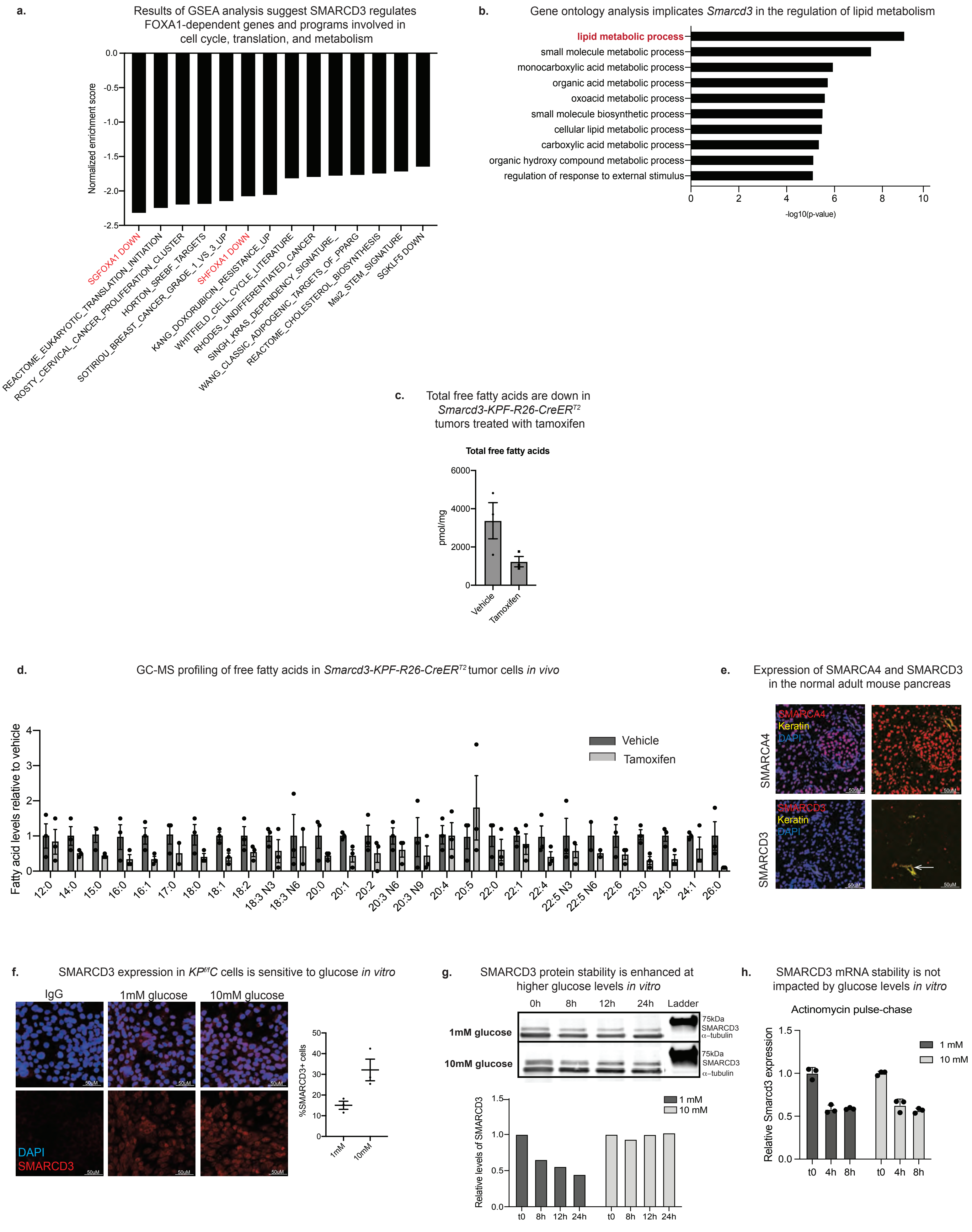

SUPPLEMENTARY FIGURE 5

Supplementary Figure 5: SMARCD3 regulates transcriptional networks implicated in lipid metabolism

a. Genes down-regulated by *Smarcd3* knockdown are enriched within FOXA1-regulated gene sets. Gene set enrichment analysis (GSEA) on our RNA-seq dataset revealed a significant enrichment for two FOXA1-regulated gene sets within genes down-regulated by *Smarcd3* knockdown (fdr<0.15).

b. Gene ontology analysis implicates *Smarcd3* in the regulation of lipid metabolism. Gene ontology (GO) analysis of RNA-seq genes down-regulated by *Smarcd3* knockdown were enriched (Fisher's exact test) for GO annotations in metabolism; lipid metabolic process was the most significantly enriched GO term.

c. Total free fatty acid levels are reduced in tamoxifen-treated *Smarcd3$^{f/f}$-KPF-R26-CreER^{T2}* tumors. EpCAM+ tumor cells from *Smarcd3$^{f/f}$-KPF-R26-CreER^{T2}* tumors treated with vehicle or tamoxifen were isolated for free fatty acid analysis by GC-MS (n=3 tumors, mean ± SEM).

d. GC-MS profiling of free fatty acids in *Smarcd3$^{f/f}$-KPF-R26-CreER^{T2}* tumors EpCAM+ tumor cells from *Smarcd3$^{f/f}$-KPF-R26-CreER^{T2}* tumors treated with vehicle or tamoxifen were isolated for free fatty acid analysis by GC-MS; all species are shown here (n=3 tumors per group, mean ± SEM).

e. Expression of SMARCA4 and SMARCD3 in the normal adult mouse pancreas. SMARCA4 or SMARCD3 (red) expression in epithelium (pan-keratin+, yellow) of adult mouse pancreatic tissue (8 weeks old); nuclei stained with DAPI (blue); pancreatic ductal structure denoted with white arrow (representative from n=2 mice, scale bar = 50um).

f. SMARCD3 expression is sensitive to glucose. *KP$^{f/f}$C* cells were cultured to >75% confluency in 2D on in media containing 1mM or 10mM glucose; slides were analyzed for the frequency of nuclear (DAPI, blue) SMARCD3+ (red) cells by immunofluorescence (representative images from n=2 replicates at n=3 frames, mean ± SEM, scale bar = 50uM).

g. SMARCD3 protein stability is enhanced at higher glucose levels *in vitro. KP$^{f/f}$C* cells were cultured in 1mM or 10mM glucose for 24 h; media was then refreshed with 1mM or 10mM glucose media containing 100ug/mL cycloheximide. Cells were collected at 0h, 8h, 12h, and 24h and SMARCD3 levels were analyzed by western blot (α-tubulin loading control, representative, n=2).

h. SMARCD3 mRNA stability is not impacted by glucose levels *in vitro. KP$^{f/f}$C* cells were cultured in 1mM or 10mM glucose for 24 h; media was then refreshed with 1mM or 10mM glucose media containing 10ng/mL actinomycin. Cells were collected at 0h, 4h, and 8h and *Smarcd3* transcript levels were analyzed by qPCR (expression normalized to b2M). Representative of n=3 biological replicates at n=3, mean ± SEM.

All source data provided in the Source Data file.

Supplementary Table 1

Lipid metabolism-associated genes annotated within all lipid-associated subnetwork hubs

Function	Genes
Lipid transport/storage	Vldlr, Ldlr, Fabp4, Dgat2, Mogat2
Metabolic regulation	Pparg, Srebf2
Cholesterol metabolism	Lss, Hmgcr, Soat2, Pmvk, Fdps, Hmgcs1, Idi1, Mvd, Mvk, Nsdhl, Sqle, Ephx2, Dhcr7
Prostaglandin synthesis	Ptgs1, Ptgs2, Ptges
Fatty acid synthesis	Scd1, Acss1, Acss2, Elovl1, Elovl7
Beta oxidation	Ech1, Acads, Acat1, Acat2, Decr1, Echs1, Eci1

Supplementary Table 2: Antibodies

Antibody target	Application	Source	Catalogue #	Dilution
EpCAM-APC	FACS, mouse tissue	eBioscience	17-5791-82	2ug/106 cells
CD45-PeCy7	FACS, mouse tissue	eBioscience	25-0451-82	2ug/106 cells
CD31-PE	FACS, mouse tissue	eBioscience	12-0311-82	2ug/106 cells
PDGFRa-BV421	FACS, mouse tissue	BD Biosciences	566293	2ug/106 cells
CD133-APC	FACS, mouse tissue	eBioscience	17-1331-81	2ug/106 cells
BrDU-APC	FACS, mouse tissue	BD Biosciences	552598	2ug/106 cells
CD133-PE	FACS, mouse tissue	BD Biosciences	12-1331-82	2ug/106 cells
AnnexinV-APC	FACS, mouse tissue	eBioscience	88-8007-72	2ug/106 cells
EpCAM-PE	FACS, human tissue	ThermoFisher	12-9326-42	2ug/106 cells
CD133-BV421	FACS, human tissue	BD Biosciences	566598	2ug/106 cells
CD133-APC	FACS, human tissue	Milltenyi	130-113-746	2ug/106 cells
α-tubulin	Western blot	Abcam	ab7291	1:10,000
SMARCD2	Western blot	Abcam	ab221168	1:500
SMARCD1	Western blot	BD Biosciences	611728	1:500
SMARCD3	Western blot	Abcam	ab204745	1:1,000
igG	IP-Western, IP-MS	Cell Signaling Technologies	2729S	1:100 (IP)
SMARCA4	IP-Western, IP-MS, CoIP-Western, ChIP	Abcam	ab110641	1:100 (IP), 1:1000 (Western)
BRD9	IP-Western	Active Motif	61537	1:100 (IP), 1:1000 (Western)
ARID1A	IP-Western	Santa Cruz	sc-32761	1:100 (IP), 1:1000 (Western)
SMARCD1	IP-Western	Santa Cruz	sc-135843	1:100 (IP), 1:1000 (Western)
SMARCD3	IP-Western	Cell Signaling Technologies	62265	1:100 (IP), 1:1000 (Western)
FLAG	CoIP-Western	ThermoFisher	MA1-91878	1:1,000
V5	CoIP-Western	ThermoFisher	R960-25	1:1,000
Biotinylated anti-rabbit	Immunofluorescence	Millipore sigma	AP187B	1:200
SMARCD3	Immunofluorescence, PLA (mouse tissue)	Abcam	ab204745	1:100
SMARCD3	Immunofluorescence (human tissue)	Aviva Systems Biology	ARP35652_P050, QC20007-43594	1:100-1:400
pan-cytokeratin	Immunofluorescence	abcam	ab8068	1:15
FOXA1	PLA	ThermoFisher	PA5-18168	1:100
SMARCA4	PLA	ThermoFisher	A303-877A	1:500
H3K27ac	ChIP	abcam	ab4729	1:100
ARID1A	ChIP	Cell Signaling Technologies	12354	1:100
FOXA1	ChIP	Abcam	ab170933	1:100
KLF5	ChIP	Abcam	ab24331	1:100
H3K4me	ChIP	Abcam	ab8895	1:100
H3K4me3	ChIP	Millipore sigma	05-745	1:100
AlexaFluor-568 anti-rabbit	Immunofluorescence	Thermo Fisher	A11036	1:500
AlexaFluor-488 anti-mouse	Immunofluorescence	Thermo Fisher	A11001	1:500
AlexaFluor-647 anti-mouse	Immunofluorescence	Thermo Fisher	A-21240	1:500
AlexaFluor-488 anti-rabbit	Immunofluorescence	Thermo Fisher	A-11008	1:500
AlexaFluor-568 anti-mouse	Immunofluorescence	Thermo Fisher	A-11004	1:500

Supplementary Table 3: Reagents, plasmids, software, and algorithms

**Commerical reagents**	**Source**
HBSS	Gibco, Life Technologies
DMEM	Gibco, Life Technologies
Pen/Strep	Gibco, Life Technologies
Non-essential amino acids	Gibco, Life Technologies
FC block	BD Bioscience
MEM	Gibco, Life Technologies
Gey's balanced salt solution	Sigma
Collagenase P	Roche
Dnase I	Roche
Rho Kinase inhibitor Y-27632	SelleckChem
RBC lysis buffer	eBioscience
Matrigel	BD Bioscience
DMEM-F12	Gibco, Life Technologies
B27 supplement	Gibco, Life Technologies
B-mercaptoethanol	Gibco, Life Technologies
N2 supplement	Gibco, Life Technologies
mEGF	Gibco, Life Technologies
bFGF2	Gibco, Life Technologies
3D CellTiterGlo Assay	Promega
Celecoxib	SelleckChem
Lovastatin	SelleckChem
Etomoxir	SelleckChem
TOFA	SelleckChem
CAY-10566	SelleckChem
Fatostatin	SelleckChem
Advanced DMEM-F12	ThermoFisher
HEPES pH 7.2-7.5	ThermoFisher
Glutamax	ThermoFisher
Primocin	Invivogen
Nicotinamide	Sigma
N-acetyl cysteine	Sigma
mNoggin	Peprotech
hEGF	Peprotech
hFGF	Peprotech
hGastrin	Tocris
A83-01	Tocris
Cell Recovery Solution	Corning
TrypLE Express	ThermoFisher
BrDU flow cytometry kit	BD Bioscience
Annexin V apoptosis kit	eBioscience
Tamoxifen	Sigma
Corn oil	Sigma
Gemcitabine	Sigma
Pronase	Sigma
Propidium Iodide	Life Technologies
4X Laemmli buffer	Biorad
4-15% precast Mini-PROTEAN TGX gel	Biorad
Odyssey buffer	Li-cor
FLAG magnetic beads	ThermoFisher
FLAG peptide	ThermoFisher
10% formalin	Millipore Sigma
Human PDAC TMA	US Biomax, Inc
Citrate Buffer	eBioscience
Triton-X 100	Sigma
Normal goat serum	Fisher Scientific
4% paraformaldehyde	Fisher Scientific
Bovin serum albumin	Invitrogen
DAPI	Molecular probes
DuoLink PLA detection kit, red	Millipore Sigma
Glucose solution	ThermoFisher
Actinomycin	Cayman chemical
Cycloheximide	Cayman chemical
Disuccinimidyl glutarate	Sigma
Protein A+G Dynabeads	Invitrogen
NuGen Ovation Ultralow Library System V2	Tecan
Nuclear Co-IP kit	Active Motif

Supplementary Table 3 cotinued: Reagents, plasmids, software, and algorithms

Plasmids

FG12 lentiviral vector	Qin et al. 2003[1]
lentiCRISPRv2	Sanjana et al. 2014[2]
SMARCD3 GFP overexpression vector	Albini et al. 2013, 2014[3,4]

Software and algorithms

10X Genomics CellRanger v3.0	Zheng et al. 2017[5]
Seurat v3.1 R Package	Satija et al. 2015[6]
HOMER v4.8	Heinz et al. 2010[7]
GSEA desktop 4.0.3	Subramanian et al. 2005[8]
STRING interactome 1.5.1	Szklarczyk et al. 2015[9]
Cytoscape v3.8.2	Shannon et al. 2003[10]
clusterMaker2 2.2	Morris et al. 2011[11]
QuPath v0.2.3	Bankhead et al. 2017[12]
ImageJ version 1.50i	Schneider et al. 2012[13]
FlowJo v10.5.3	Beckton Dickson
Graphpad PRISM v8.2.0	GraphPad
Umap 0.3.8	McInnes, L., & Healy, J. 2018[14]
STAR v2.5	Dobin et al. 2013[15]
DESEQ2 3.15	Love et al. 2014[16]
FACS Diva v6.1.3.	Beckton Dickson
Scorenado	Github

Supplementary Table 4: Oligonucleotide sequences

shRNA oligos

Mouse	Target sequence
shControl	GCAGTTATCTGGAAGATCAGG
shSmarcd3-1	TGCGCCTTTATATCTCCAATA
shSmarcd3-2	ACATGGACCTCCTAGCATTTG
shKlf4	AGTTGGACCCAGTATACATTC
shZic5	ACTTGCCACCGGGTCTAATTA
shSox9	GCGACGTCATCTCCAACATTG
shMeis2	GACGGGCTCATCGAGACAATT
shZbtb12	GAACGCCCTTAGCCAGTTCAT
shOct4	CAAGTTGGCGTGGAGACTTTG
shFoxa1-1	TAGTTCCTGCAGGGCTTATTT
shFoxa1-3	GCGCTGCAGTACTCTCCTTAT

Human

shControl	GCAGTTATCTGGAAGATCAGG
shSmarcd3-1	AGACGGCGTGCTATGACATTG
shSmarcd3-2	AGCGGAAGCTGCGACTCTATA
shSmarcd3-3	GACAAGTATTTCCAGCAGATT

CRISPR guides

NT1	CACCGGCGAGGTATTCGGCTCCGCG
sgSmarcd3	GCCGCGGACGAAGTTGCCGGAGG
sgMeis2	TCTTCCAGTAAAACTCCGCGAGG
sgHdac11	CTGGAGGGAGACCGCCTCGGGGG
sgHdac7	GTGCCCCGCCCAGCCTTCGGAGG
sgElf3	GACCTCAGACAAGATCCCAAAGG

**qPCR primers**	F	R
mouse Smarcd3	AGGCTTACATGGACCTCCTAG	CATCAGAGTCTTCCGCATCAG
human Smarcd3	GGAGCCGCAGTGCCAAGA	TAAGCCTGGGACTCGGGGA

Supplementary References

1. Qin, X. F., An, D. S., Chen, I. S. Y. & Baltimore, D. Inhibiting HIV-1 infection in human T cells by lentiviral-mediated delivery of small interfering RNA against CCR5. *Proc Natl Acad Sci U S A* **100**, 183–188 (2003).
2. Sanjana, N. E., Shalem, O. & Zhang, F. Improved vectors and genome-wide libraries for CRISPR screening. *Nature Methods* vol. 11 783–784 (2014).
3. Albini, S. & Puri, P. L. Generation of myospheres from hESCs by epigenetic reprogramming. *Journal of Visualized Experiments* **88**, 51243 (2014).
4. Albini, S., Coutinho, P., Malecova, B., Giordani, L., Savchenko, A., Forcales, S.V. and Puri, P.L. Epigenetic Reprogramming of Human Embryonic Stem Cells into Skeletal Muscle Cells and Generation of Contractile Myospheres. *Cell Reports* **3**, 661–670 (2013).
5. Zheng, G. X. Y. *et al.* Massively parallel digital transcriptional profiling of single cells. *Nature Communications* **8**, 1–12 (2017).
6. Satija, R., Farrell, J. A., Gennert, D., Schier, A. F. & Regev, A. Spatial reconstruction of single-cell gene expression data. *Nature Biotechnology* **33**, 495–502 (2015).
7. Heinz, S., Benner, C., Spann, N., Bertolino, E., Lin, Y.C., Laslo, P., Cheng, J.X., Murre, C., Singh, H. and Glass, C.K. Simple Combinations of Lineage-Determining Transcription Factors Prime cis-Regulatory Elements Required for Macrophage and B Cell Identities. *Molecular Cell* **38**, 576–589 (2010).
8. Subramanian, A., Tamayo, P., Mootha, V.K., Mukherjee, S., Ebert, B.L., Gillette, M.A., Paulovich, A., Pomeroy, S.L., Golub, T.R., Lander, E.S. and Mesirov, J.P. Gene set enrichment analysis: a knowledge-based approach for interpreting genome-wide expression profiles. *Proc Natl Acad Sci U S A* **102**, 15545–15550 (2005).
9. Szklarczyk, D., Franceschini, A., Wyder, S., Forslund, K., Heller, D., Huerta-Cepas, J., Simonovic, M., Roth, A., Santos, A., Tsafou, K.P. and Kuhn, M. STRING v10: Protein-protein interaction networks, integrated over the tree of life. *Nucleic Acids Research* **43**, D447–D452 (2015).
10. Shannon, P., Markiel, A., Ozier, O., Baliga, N.S., Wang, J.T., Ramage, D., Amin, N., Schwikowski, B. and Ideker, T. Cytoscape: A software Environment for integrated models of biomolecular interaction networks. *Genome Research* **13**, 2498–2504 (2003).
11. Morris, J.H., Apeltsin, L., Newman, A.M., Baumbach, J., Wittkop, T., Su, G., Bader, G.D. and Ferrin, T.E. ClusterMaker: A multi-algorithm clustering plugin for Cytoscape. *BMC Bioinformatics* **12**, (2011).
12. Bankhead, P., Loughrey, M.B., Fernández, J.A., Dombrowski, Y., McArt, D.G., Dunne, P.D., McQuaid, S., Gray, R.T., Murray, L.J., Coleman, H.G. and James, J.A. QuPath: Open source software for digital pathology image analysis. *Scientific Reports* **7**, 1–7 (2017).
13. Schneider, C. A., Rasband, W. S. & Eliceiri, K. W. NIH Image to ImageJ: 25 years of image analysis. *Nature Methods* vol. 9 671–675 (2012).
14. McInnes, L., & Healy, J. (2018). UMAP: Uniform Manifold Approximation and Projection for Dimension Reduction. *ArXiv e-prints.*
15. Dobin, A., Davis, C.A., Schlesinger, F., Drenkow, J., Zaleski, C., Jha, S., Batut, P., Chaisson, M. and Gingeras, T.R. STAR: ultrafast universal RNA-seq aligner. *Bioinformatics*, *29*(1), pp.15-21 (2013).
16. Love, M.I., Huber, W. and Anders, S. Moderated estimation of fold change and dispersion for RNA-seq data with DESeq2. *Genome biology*, *15*(12), pp.1-21 (2014).
