# Supplementary material for: Smarcd3 is an epigenetic modulator of the metabolic landscape in pancreatic ductal adenocarcinoma
Source: Nat Commun. 2023 Jan 18;14:292. doi: 10.1038/s41467-023-35796-7 (PMC9849267; doi:10.1038/s41467-023-35796-7)
Supplement: Supplementary file 2 — Description of Additional Supplementary Files [file 41467_2023_35796_MOESM2_ESM.pdf]

# Description of additional supplementary files

Supplementary Data 1:
Node genes within each functionally annotated subnetwork hub. Related to Figure 5d.

Supplementary Data 2:
Additional specific information on mice used for *in vivo* studies
