# Supplementary material for: Smarcd3 is an epigenetic modulator of the metabolic landscape in pancreatic ductal adenocarcinoma
Source: Nat Commun. 2023 Jan 18;14:292. doi: 10.1038/s41467-023-35796-7 (PMC9849267; doi:10.1038/s41467-023-35796-7)
Supplement: Supplementary file 5 — Reporting Summary [file 41467_2023_35796_MOESM5_ESM.pdf]

Corresponding author(s): Dr. Tannishtha Reya

Last updated by author(s): 11/14/2022

Reporting Summary

Nature Research wishes to improve the reproducibility of the work that we publish. This form provides structure for consistency and transparency in reporting. For further information on Nature Research policies, see our Editorial Policies and the Editorial Policy Checklist.

Statistics

For all statistical analyses, confirm that the following items are present in the figure legend, table legend, main text, or Methods section.

n/a	Confirmed	
☐	☒	The exact sample size (*n*) for each experimental group/condition, given as a discrete number and unit of measurement
☒	☐	A statement on whether measurements were taken from distinct samples or whether the same sample was measured repeatedly
☐	☒	The statistical test(s) used AND whether they are one- or two-sided<br>*Only common tests should be described solely by name; describe more complex techniques in the Methods section.*
☒	☐	A description of all covariates tested
☐	☒	A description of any assumptions or corrections, such as tests of normality and adjustment for multiple comparisons
☐	☒	A full description of the statistical parameters including central tendency (e.g. means) or other basic estimates (e.g. regression coefficient) AND variation (e.g. standard deviation) or associated estimates of uncertainty (e.g. confidence intervals)
☐	☒	For null hypothesis testing, the test statistic (e.g. *F*, *t*, *r*) with confidence intervals, effect sizes, degrees of freedom and *P* value noted<br>*Give P values as exact values whenever suitable.*
☒	☐	For Bayesian analysis, information on the choice of priors and Markov chain Monte Carlo settings
☒	☐	For hierarchical and complex designs, identification of the appropriate level for tests and full reporting of outcomes
☒	☐	Estimates of effect sizes (e.g. Cohen's *d*, Pearson's *r*), indicating how they were calculated

Our web collection on statistics for biologists contains articles on many of the points above.

Software and code

Policy information about availability of computer code

Data collection	FACS Diva v6.1.3 used to acquire Flow cytometry data
Data analysis	All software used for data analysis is described in the Methods section in detail and in Supplementary Table 3. Briefly: ImageJ v1.50i was used for image analysis, FlowJo v10.5.3 was used to analyze all FACS data, GraphPad Prism 8.2.0 was used for statistical analysis (T-cests, ANOVA, linear regression), tumor tissue area and tumor cell number analysis by histology was done using QuPath 0.2.3, Scorenado was used to analyze clinically annotated human PDAC tissue microarray, 10X Genomics Cell Ranger v3.0, Seurat v3.1, and UMAP 0.3.8 were used to analyze and plot human PDAC scRNAseq data, STAR v2.5 was used to align mouse pancreatic cancer RNA-seq and ChIP-seq data, HOMER v4.8 was used to process ChIP-seq alignment files, binding enrichment, and differential RNA-seq expression using DESEQ2 3.15, Gene Set Enrichment Analysis desktop 4.0.3 was used for GSEA analysis of RNA-seq data using Bioconductor GSVA C2, C6, and C7 BroadSets gene set collections, Cytoscape v.3.8.2 desktop was and the GLay clustering algorithm (clusterMaker2 v2.2) were used to construct the RNA-seq network using the mouse STRING interactome (1.5.1), STRING functional enrichment was used to annotate network clusters within Cytoscape. Scorenado for TMA scoring is available on github: https://github.com/digitalpathologybern/scorenado.

For manuscripts utilizing custom algorithms or software that are central to the research but not yet described in published literature, software must be made available to editors and reviewers. We strongly encourage code deposition in a community repository (e.g. GitHub). See the Nature Research guidelines for submitting code & software for further information.

Data

Policy information about availability of data

All manuscripts must include a data availability statement. This statement should provide the following information, where applicable:

- Accession codes, unique identifiers, or web links for publicly available datasets
- A list of figures that have associated raw data
- A description of any restrictions on data availability

The KPf/fC RNA-seq and H3K27-acetyl, H3K4me, H3K4me3, ARID1A, KLF5, FOXA1 and SMARCA4 ChIP-seq data generated in this study have been deposited in the GEO database under accession code GSE168490 (https://www.ncbi.nlm.nih.gov/geo/query/acc.cgi?&acc=GSE168490). The processed RNA-seq data are available also available under accession code GSE168490 (https://www.ncbi.nlm.nih.gov/geo/query/acc.cgi?&acc=GSE168490).
The IP-MS data generated in this study have been deposited in MassIVE under accession MSV000090744 (https://massive.ucsd.edu/ProteoSAFe/dataset.jsp?task=63becd806f68431c90cb6455e5e7ec12) and in the ProteomeXchange dataset under the accession number PXD038216 (http://proteomecentral.proteomexchange.org/cgi/GetDataset?ID=PXD038216).
The publicly available mouse Msi2+ and Msi2- primary KPf/fC RNA-seq data8 used in this study are available in the GEO databased under accession code GSE114906 (https://www.ncbi.nlm.nih.gov/geo/query/acc.cgi?acc=GSE114906).
The publicly available human PDAC scRNA-seq data40 used in this study are available at the Genome Sequence Archive under accession code GSA: CRA001160 (https://ngdc.cncb.ac.cn/gsa/browse/CRA001160).
Source data are provided with this paper. The remaining data are available within the Article, Supplementary Information or Source Data file.

Field-specific reporting

Please select the one below that is the best fit for your research. If you are not sure, read the appropriate sections before making your selection.

☒ Life sciences ☐ Behavioural & social sciences ☐ Ecological, evolutionary & environmental sciences

For a reference copy of the document with all sections, see nature.com/documents/nr-reporting-summary-flat.pdf

Life sciences study design

All studies must disclose on these points even when the disclosure is negative.

Sample size	Sample sizes for in vivo studies were determined based on previous experience and preliminary analyses, and are determined by power analyses to be the minimum number needed to achieve statistical significance for the work proposed (G*Power, Faul et al. 2007, where power is 90% and p value<0.05).
Data exclusions	In a few cases, data points were excluded from the analysis of KPf/fC tumors if these data points were identified as an outlier by statistical measures. In each case where an outlier has been excluded, this is specifically noted in each figure legend along with the specific statistical test that has been used.
Replication	The level of replication for each in vitro and in vivo study is noted in the figure legends for each figure and described in detail in the Methods section, but will be briefly summarized here. However, to summarize briefly, in vitro sphere or colony formation studies were conducted with n=3 independent wells per cell line across two independent shRNA of n=3 wells; the majority of these experiments were additionally completed in >2 independently derived cell line, n=3 wells per shRNA. Because material was limited, PDX organoids treated with shRNA were plated in n=3-4 wells per experiment, for one experiment each using two independent PDX organoid lines. Flank shRNA studies were conducted three times using independent cell lines, with n=3-4 tumors per group in each experiment. Analysis of midpoint (7-8 weeks old) KPf/fC tumors was conducted with n=5-16 mice per group. Secondary syngeneic transplants were conducted with n=3-4 independent tumors per group, transplanted into n=2-4 littermate recipients each. Survival studies in KPf/fC mice plus and minus gemcitabine treatment were conducted with n=6-10 mice per group. Flank KPF + adCre and KPF-R26-CreERT2 tamoxifen treated transplants were conducted in 3 biological replicates at n=3-5 tumors per group. Autochthonous tumor studies were completed in n=4-5. Tumor initiation studies in the autochthonous KC model were conducted with n=3-9 mice for all Cre systems used. 3 independent PDX tumors were used for shRNA studies in vivo, one PDX sample was used for one experiment while the other two were completed in duplicate for a total of n=4-5 per shRNA for 2 independent shRNA. RNA-seq in KPf/fC cells was run in triplicate, H3K27-acetyl ChIP-seq was run in duplicate, and one ChIP each was run for H3K4me, H3K4me3, SMARCA4, ARID1A, FOXA1, and KLF5 ChIP-seq.
Randomization	Randomization for in vivo studies is described in detail in the Methods section. Briefly, for all in vivo studies using authochthonous mouse models, no sexual dimorphism was noted in all mouse models. Therefore, males and females of each strain were equally used for experimental purposes and both sexes are represented in all data sets; littermates of the same sex were randomized into experimental groups when applicable or possible based on available mice. Mice were chosen for transplants and analysis of primary tumors at random in the order in which mice of the correct genotype were born. All flank transplants were measured, binned by size, and enrolled into two treatment arms in order of size. For secondary syngeneic transplants of primary KPf/fC cells, male and female littermate recipients were used equivalently when possible given limited number of littermates of the appropriate genotype. The sex, age, and number of all mice for all experiments are detailed in Supplementary Table 3.
Blinding	For sphere-forming and in vitro growth assays, specific wells were kept blinded during counting and analysis.<br>For immunofluorescence analysis, tissue sections were de-identified when possible until after analysis. Tissue section genotype as well as disease model were blinded for pathological analysis of initiation models. For pathological analysis of tumor initiation models, pathologist was blinded to genotype of mice for analysis. The investigators were not blinded during outcome assessment for other experiments since data acquisition and analysis were done using indicated software.

Reporting for specific materials, systems and methods

We require information from authors about some types of materials, experimental systems and methods used in many studies. Here, indicate whether each material, system or method listed is relevant to your study. If you are not sure if a list item applies to your research, read the appropriate section before selecting a response.

Materials & experimental systems

n/a	Involved in the study
	☒ Antibodies
	☒ Eukaryotic cell lines
☒	Palaeontology and archaeology
	☒ Animals and other organisms
☒	Human research participants
☒	Clinical data
☒	Dual use research of concern

Methods

n/a	Involved in the study
	☒ ChIP-seq
	☒ Flow cytometry
☒	MRI-based neuroimaging

Antibodies

Antibodies used

For FACS: anti-mouse EpCAM-APC (eBioscience, #17-5791-82), anti-mouse CD45-PeCy7 (eBioscience, #25-0451-82), anti-mouse CD31-PE (eBioscience, #12-0311-82), anti-mousePDGFR-BV421 (BD Bioscience, 566293, anti-mouse CD133-PE (eBioscience, #12-1331-82), anti-mouse CD133-APC (eBioscience, #17-1331-81), anti-mouse BrDU-APC (BD Bioscience, 552598), anti-mouse AnnexinV-APC (eBioscience, #88-8007-72), anti-human EpCAM-PE (ThermoFisher #12-9326-42), anti-human CD133-BV421 (BD Biosciences, #566598) or anti-human CD133-APC (Miltenyi #130-113-746). All FACS antibodies used at 2ug/10^6 cell dilution. For Western Blot: anti-α-tubulin (Abcam, ab7291, 1:10,000), anti-SMARCD2 (Abcam, ab221168, 1:500), anti-SMARCD1 (BD Biosciences, 611728, 1:500), anti-SMARCD3 (Abcam, ab204745, 1:1000). For IP-Western blot and IP-Mass spectrometry: anti-IgG (Cell Signaling, 2729S, 1:100), anti-SMARCA4 (Abcam, ab110641, 1:100), anti-BRD9 (Active Motif, 61537, 1:100), anti-ARID1A (Santa Cruz, sc-32761, 1:100), anti-SMARCD1 (Santa Cruz, sc-135843, 1:100), anti-SMARCD3 (Cell Signaling, 62265, 1:100), anti-FLAG (ThermoFisher, MA1-91878, 1:1000), anti-V5 (ThermoFisher, R960-25, 1:1,000). For immunofluorescence (IF) and proximity ligation assay: anti-mouse SMARCD3 (Abcam ab204745, 1:100), anti-human SMARCD3 (Aviva Systems Biology, ARP35652_P050, QC20007-43594, 1:100), anti-Keratin (Abcam, ab8068, 1:15), anti-FOXA1 (Thermo Fisher, PA5-18168, 1:100), anti-SMARCA4 (Thermo Fisher, A303-877A, 1:500). For ChIP-seq: anti-H3K27ac (Abcam, ab4729), anti-ARID1A (Cell Signaling, 12354), anti-FOXA1 (Abcam, ab170933), anti-KLF5 (Abcam, ab24331), anti-H3K4me (Abcam, ab8895), anti-H3K4me3 (Millipore-Sigma, 05-745). All ChIP antibodies were used at 1:100 dilution.
All specific antibody catalogue numbers and dilutions are now detailed in Supplementary Table 2.

Validation

EpCAM-APC (eBioscience, #17-5791-82): "This G8.8 antibody has been tested by flow cytometric analysis of the TE-71 cell line". CD45-PeCy7 (eBioscience, #25-0451-82): "This 30-F11 antibody has been tested by flow cytometric analysis of mouse splenocytes". CD31-PE (eBioscience, #12-0311-82): "This Antibody was verified by Cell treatment to ensure that the antibody binds to the antigen stated. The 390 antibody has been tested by flow cytometric analysis of mouse thymocytes and splenocytes". PDGFR-BV421 (BD Bioscience, 566293): "Reactivity: Mouse (QC Testing); Application: Flow cytometry (Routinely Tested)". CD133-PE (eBioscience, #12-1331-82): "This 13A4 antibody has been tested by flow cytometric analysis of mouse bone marrow cells.". CD133-APC (eBioscience, #17-1331-81): "This 13A4 antibody has been tested by flow cytometric analysis of mouse bone marrow cells". BrDU-APC (BD Bioscience, 552598): No manufacturer statement on validation. AnnexinV-APC (eBioscience, #88-8007-72): No manufacturer statement on validation. EpCAM-PE (ThermoFisher, #12-9326-42): "This 1B7 antibody has been pre-titrated and tested by flow cytometric analysis of A549 human lung carcinoma cells". CD133-BV421 (BD Biosciences, #566598): "Reactivity: Human (QC Testing); Application: Flow cytometry (Routinely Tested), Immunofluorescence, Immunohistochemistry-frozen (Tested During Development)". CD133-APC (Miltenyi, #130-113-746): Validated by manufacturer in flow cytometry. α-tubulin (Abcam, ab7291): Validated by manufacturer in western blot using HeLa, PC12, SV40LT-SMC, NIH/3T3 cell, rat liver, and rat heart lysate. SMARCD2 (Abcam, ab221168): Validated by manufacturer in ICC/IF using MCF7 cells; we validated for western blot in-house using knockdown in mouse pancreatic cancer cells. SMARCD1 (BD Biosciences, 611728): "Mouse, Rat, Dog, Chicken (Tested in Development), Application: Western blot (Routinely Tested)"; we validated for western blot in-house using knockdown in mouse pancreatic cancer cells. SMARCD3 (Abcam, ab204745): Validated by manufacturer in IHC-P, ICC/IF, WB; we validated for western blot in-house using knockdown in mouse pancreatic cancer cells; we validated for IF on knockout mouse pancreatic cancer tumor tissue. anti-IgG (Cell Signaling, 2729S): "Immunoprecipitation of 4E-BP2 from C2C12 cell extracts using Normal Rabbit IgG #2729 (lane 2) or 4E-BP2 Antibody #2845 (lane 3). Lane 1 is 10% input. Western blot analysis was performed using 4E-BP2 Antibody #2845". anti-SMARCA4 (Abcam, ab110641): Knockout validated, validated by manufacturer in WB, IP, IHC-P, ICC/IF, Flow Cyt. anti-BRD9 (Active Motif, 61537): "Validated Applications: ChIP: 5 - 10 µl per ChIP, ChIP-Seq: 5 - 10 µl each, IP: 10 µl per IP, WB: 1:500 - 1:2,000 dilution, IHC (FFPE): 1:100 dilution; ChIP-Seq validation was performed by Active Motif's Epigenetics Services and the complete data set is available in the UCSC Genome Browser". anti-ARID1A (Santa Cruz, sc-32761): Validated by manufacturer in WB, IF, IHC. anti-SMARCD1 (Santa Cruz, sc-135843): Validated by manufacturer in WB, IF. anti-SMARCD3 (Cell Signaling, 62265): Validated by manufacturer in WB, IP using HeLa, 3T3, H-4-II-E, and COS-7 cells. anti-FLAG (ThermoFisher, MA1-91878): Validated by manufacturer in IF, WB, and IP; "This Antibody was verified by Cell treatment to ensure that the antibody binds to the antigen stated". anti-V5 (ThermoFisher, R960-25): Validated by manufacturer in IF and WB; "This Antibody was verified by Relative expression to ensure that the antibody binds to the antigen stated". anti-human SMARCD3 (Aviva Systems Biology, ARP35652_P050, QC20007-43594): Validated by manufacturer in WB, IHC; we validated in-house by testing against knockout mouse pancreatic tumors by IF and WB. anti-Keratin (Abcam, ab8068): Validated by manufacturer in Flow Cyt, WB, ICC/IF. anti-FOXA1 (Thermo Fisher, PA5-18168): Validated by manufacturer in WB, IHC, Flow Cyt. anti-SMARCA4 (Thermo Fisher, A303-877A): validated by manufacturer in WB, IHC, IP. anti-H3K27ac (Abcam, ab4729): Validated by manufacturer in ICC/IF, WB, IHC-P, ChIP, PepArr. anti-ARID1A (Cell Signaling, 12354): Validated by manufacturer in WB, IHC, ChIP. anti-FOXA1 (Abcam, ab170933): Validated by manufacturer in ChIP, Flow Cyt (Intra),

WB, IHC-P, ICC/IF. anti-KLF5 (Abcam, ab24331): Validated by manufacturer in IHC-P, WB. anti-H3K4me (Abcam, ab8895): Validated by manufacturer in ChIP, WB, IHC-P/IF. anti-H3K4me3 (Millipore-Sigma, 05-745): Validated by manufacturer in WB, ChIP.

Eukaryotic cell lines

Policy information about cell lines

Cell line source(s)	FG (COLO-357) human pancreatic cancer cells were provided by Dr. Andrew Lowy.Mouse primary pancreatic cancer cell lines were established from end-stage wild-type KPf/fC and Msi2-GFP-KPf/fC (9-12 weeks of age), KPC (16-20 weeks of age), Smarcd3f/f-KPF (10-15 weeks of age) mice by isolating primary tumors, dissociating to a single cell suspension and plating in 2D culture. At the first passage, cells were collected and EpCAM-APC+ tumor cells were sorted and re-plated for at least one additional passage. Patient-derived organoid lines were derived by isolating end-stage patient-derived xenograft tumors and dissociating to single cell and plating in a matrigel dome covered in human organoid growth media.
Authentication	FG human pancreatic cancer cells were previously derived from a PDAC metastasis and validated by Morgan et al. 1980. To evaluate any cellular contamination and validate the epithelial nature of primary mouse pancreatic cancer cell lines, cells were analyzed by flow cytometry again at the second passage for markers of blood cells, endothelial cells, and fibroblasts (PDGFR-PacBlue, Biolegend). Patient-derived organoid lines were passaged to select for cancer cells as previously described (Baker et al. 2017) and checked for EpCAM expression to validate their epithelial nature. We have not authenticated any cell lines using STR fingerprinting.
Mycoplasma contamination	All cell lines were regularly tested for the presence of mycoplasma and verified to be negative.
Commonly misidentified lines (See ICLAC register)	None

Animals and other organisms

Policy information about studies involving animals; ARRIVE guidelines recommended for reporting animal research

Laboratory animals	All mice used were between 6 and 16 weeks old and both male and female mice were used. LSL-Kras G12D (KrasG12D/+) mice, B6.129S4-Krastm4Tyj/J (Stock No: 008179), p53flox/flox (p53f/f) mice, B6.129P2- Trp53tm1Brn/J (Stock No: 008462), R26-CreERT2 mice, B6.129-Gt(ROSA)26Sortm1(cre/ERT2)Tyj/J (Stock No: 008463), Ptf1aCRE-ERTM, Ptf1atm2(cre/ESR1)Cvw/J (Stock No: 019378), and Sox9-CreERT2, Tg(Sox9-cre/ERT2)1Msan/J (Stock No: 018829) mice were purchased from The Jackson Laboratory. Msi2eGFP/+ (Msi2-GFP) reporter mice were generated as previously described(Fox et al. 2016); all of the reporter mice used in experiments were heterozygous for the Msi2 allele. Dr. Chris Wright provided p48-Cre (Ptf1a-Cre) mice. LSL-R172H mutant p53 (p53R172H/+), Trp53R172H mice were provided by Dr. Tyler Jacks (JAX Stock No: 008183). Dr. Benoit Bruneau generated Smarcd3f/f mice; mice were provided by Dr. Lorenzo Puri. Dr. Dieter Saur provided Pdx-FlpOKI (Pdx-Flp), p53frt/frt, and FSF-KrasG12D/+ mice. Immune compromised NOD/SCID (NOD.CB17-Prkdcscid/J, Stock No: 001303) and NSG (NOD.Cg-PrkdcscidIL2rgtm1Wji/SzJ, Stock No: 005557) mice were purchased from The Jackson Laboratory.
Wild animals	This study did not involve wild animals.
Field-collected samples	The study involved no field-collected samples
Ethics oversight	IAUCUC and the University of California San Diego approved our animal use protocol for all strains, experiments, and procedures outlined in these studies.

Note that full information on the approval of the study protocol must also be provided in the manuscript.

ChIP-seq

Data deposition

- [x] Confirm that both raw and final processed data have been deposited in a public database such as GEO.
- [x] Confirm that you have deposited or provided access to graph files (e.g. BED files) for the called peaks.

Data access links *May remain private before publication.*	All sequencing data has been submitted at GEO, GSE168490. Reviewer token: anqruiogprufpkb
Files in database submission	KPC_BRG1_shControl.ucsc.bedGraph.gz<br>KPC_BRG1_shsmarcd3.ucsc.bedGraph.gz<br>KPC_shControl_ARID1A.ucsc.bedGraph.gz<br>KPC_shControl_H3K27ac-Rep1.ucsc.bedGraph.gz<br>KPC_shControl_H3K27ac-Rep2.ucsc.bedGraph.gz<br>KPC_shControl_H3K4me.ucsc.bedGraph.gz<br>KPC_shControl_H3K4me3.ucsc.bedGraph.gz<br>KPC_shSmarcd3_ARID1A.ucsc.bedGraph.gz<br>KPC_shSmarcd3_H3K27ac-Rep1.ucsc.bedGraph.gz

KPC_shSmarcd3_H3K27ac-Rep2.ucsc.bedGraph.gz
KPC_shSmarcd3_H3K4me.ucsc.bedGraph.gz
KPC_shSmarcd3_H3K4me3.ucsc.bedGraph.gz
getDiffExp_Output_KPC_shControl_shSmarcd3.txt
KPC_Smarcd3_Rep3_R1_001.fastq.gz
KPC_shControl_Rep1_R1_001.fastq.gz
KPC_shControl_Rep2_R1_001.fastq.gz
KPC_shControl_Rep3_R1_001.fastq.gz
KPC_shSmarcd3_Rep1_R1_001.fastq.gz
KPC_shSmarcd3_Rep2_R1_001.fastq.gz
KPC_shControl_ARID1A_ChIP_R1.fastq.gz
KPC_shControl_BRG1_ChIP_E1_R1.fastq.gz
KPC_shControl_H3K27ac-1_E2_R1.fastq.gz
KPC_shControl_H3K27ac-2_E2_R1.fastq.gz
KPC_shControl_H3K4me3_E1_R1.fastq.gz
KPC_shControl_H3K4me_E1_R1.fastq.gz
KPC_shControl_Input_E1_R1.fastq.gz
KPC_shControl_Input_R1.fastq.gz
KPC_shControl_Input_to_histone_E1_R1.fastq.gz
KPC_shControl_Input_to_histone_E2_R1.fastq.gz
KPC_shSmarcd3_ARID1A_ChIP_R1.fastq.gz
KPC_shSmarcd3_BRG1_ChIP_E1_R1.fastq.gz
KPC_shSmarcd3_H3K27ac-1_E2_R1.fastq.gz
KPC_shSmarcd3_H3K27ac-2_E2_R1.fastq.gz
KPC_shSmarcd3_H3K4me3_E1_R1.fastq.gz
KPC_shSmarcd3_H3K4me_E1_R1.fastq.gz
KPC_shSmarcd3_Input_E1_R1.fastq.gz
KPC_shSmarcd3_Input_R1.fastq.gz
KPC_shSmarcd3_Input_to_histone_E1_R1.fastq.gz
KPC_shSmarcd3_Input_to_histone_E2_R1.fastq.gz

Genome browser session (e.g. UCSC)

no longer applicable

Methodology

Replicates

ChIP-seq for H3K4me, H3K4me3, ARID1A, and SMARCA4 were conducted in 1 replicate each; ChIP-seq for H3K27ac was completed in duplicate.

Sequencing depth

Sequencing was single-end, 50bp reads, at a sequencing depth of ~30million for these studies, >80% of reads mapped uniquely on average.

Antibodies

anti-SMARCA4 (Abcam 110641), anti-ARID1A (CST 12354), H3K4me (Abcam ab8895), H3K4me3 (Millipore 05-745), H3K27ac (Abcam ab4729).

Peak calling parameters

Reads were aligned to the mouse genome (mm10) using STAR alignment tool (v2.5). Only reads that mapped to a unique genomic location (MAPQ>10) were used for downstream analysis. HOMER123 (v4.8) was used to process alignment files to generate ChIP-seq bed files. ChIP-seq peaks for SMARCA4 and ARID1A were found by using the findPeaks program in HOMER with the parameter "-style factor" versus the appropriate ChIP input experiments as background. ChIP-seq peaks for H3K4me, H3K4me3, and H3K27ac were called using the parameter "-style histone". SMARCA4 and ARID1A peaks were called when enriched >four-fold over input and over local tag counts, with FDR 0.001 (Benjamin-Hochberg). For histone ChIP-seq, peaks within a 1000 bp range were stitched together to form regions. Differential ChIP-seq peaks were found by merging peaks from shControl and shSmarcd3 groups and called using getDifferentialPeaks with fold change 1.5, Poisson p value < 0.0001.

Data quality

To ensure data quality only reads that mapped to a unique genomic location (MAPQ>10) were used for downstream analysis.

Software

Reads were aligned to the mouse genome (mm10) using STAR alignment tool (v2.5). HOMER123 (v4.8) was used to process alignment files to generate ChIP-seq bed files. ChIP-seq peaks for SMARCA4 and ARID1A were found by using the findPeaks program in HOMER. Differential ChIP-seq peaks were found by merging peaks from shControl and shSmarcd3 groups and called using getDifferentialPeaks with fold change 1.5, Poisson p value < 0.0001.

Flow Cytometry

Plots

Confirm that:

- [x] The axis labels state the marker and fluorochrome used (e.g. CD4-FITC).
- [x] The axis scales are clearly visible. Include numbers along axes only for bottom left plot of group (a 'group' is an analysis of identical markers).
- [x] All plots are contour plots with outliers or pseudocolor plots.
- [x] A numerical value for number of cells or percentage (with statistics) is provided.

Methodology

Sample preparation

To analyze in vitro proliferation (BrdU) or cell death (Annexin V) by FACS, KPf/fC or FG were infected with shRNA and sorted 72 h later; 50,000 transduced cells were plated in a 24-well plate in 10% DMEM. For BrdU analysis, 24 h after plating, media was refreshed with media containing BrdU (BD Biosciences) as per manufacturers instructions; after an 18 h pulse in BrdU-containing media, cells were trypsinized, fixed, permeabilized, and stained with anti-BrdU-APC using the BrdU flow cytometry kit (BD Biosciences). For Annexin V analysis, cells were trypsinized and analyzed with the Annexin V apoptosis kit (eBioscience) 48 h after plating.

Mouse pancreatic tumors from mid-point KPf/fC mice, syngeneic secondary KPf/fC transplants, KPF and KPF-R26-CreERT2 transplants were dissociated and analyzed by FACS as follows. Mouse pancreatic tumors were washed in MEM (Gibco, Life Technologies) and cut into 1–2 mm pieces immediately following resection. Tumor pieces were collected into a 50 ml Falcon tube containing 10 ml Gey's balanced salt solution (Sigma), 5 mg Collagenase P (Roche), 2 mg Pronase (Roche), and 0.2 µg DNAse I (Roche). Samples were incubated for 15 minutes at 37°C, then pipetted up and down 10 times and returned to 37°C. After 15 more minutes, samples were pipetted up and down 5 times, then passed through a 100 µm nylon mesh (Corning). Red blood cells were lysed using RBC Lysis Buffer (eBioscience) and the remaining tumor cells were washed, then resuspended in HBSS (Gibco, Life Technologies) containing 2.5% FBS and 2 mM EDTA for staining, FACS analysis, and cell sorting. Analysis and cell sorting were carried out on a FACSAria III machine (Becton Dickinson), and data were analyzed with FlowJo software v.10.5.3 (Tree Star). The following rat antibodies were used: anti-mouse EpCAM-APC (eBioscience), anti-mouse CD133-PE (eBioscience, #12-1331-82), anti-mouse CD45-PE/Cy7 (eBioscience, #25-0451-82), anti-mouse CD31-PE (BD Bioscience, #12-0311-82), anti-mouse PDGFR-BV421 (BD Bioscience, 566293 (Biolegend), anti-mouse BrdU-APC (BD Biosciences, 552598), and anti-mouse Annexin-V-APC (eBioscience, #88-8007-72). Propidium-iodide (Life Technologies) was used to stain for dead cells. Msi2 expression was assessed by GFP expression in Msi2-GFP-KPf/fC mice.

Patient-derived xenograft tumors were washed in MEM (Gibco, Life Technologies) and cut into 1–2 mm pieces immediately following resection. Tumor pieces were collected into a 50 ml Falcon tube containing 10 ml Gey's balanced salt solution (Sigma), 5 mg Collagenase P (Roche), 0.2 µg DNAse I (Roche), and 10.5uM Rho Kinase inhibitor (SelleckChem, Y-27632). Samples were incubated for 10 minutes at 37°C, then pipetted up and down 10 times and returned to 37°C. After 10 more minutes, samples were pipetted up and down 5 times, then passed through a 100 µm nylon mesh (Corning). Red blood cells were lysed using RBC Lysis Buffer (eBioscience) and the remaining tumor cells were washed, then resuspended in HBSS (Gibco, Life Technologies) containing 2.5% FBS and 2 mM EDTA for staining as described above. Human tissues were stained with rat antibodies against anti-human EpCAM-PE (ThermoFisher #12-9326-42) and CD133-BV421 (BD Biosciences, #566598) or CD133-APC (Miltenyi #130-113-746).

Instrument

Analysis and cell sorting were carried out on a FACSAria III machine (BD Bioscience).

Software

FACSDiva v6.1.3 (BD Biosciences) was used to collect data and FlowJo v10.5.3 was used to analyze all FACS data.

Cell population abundance

The purity of post-sort cells was over 95% as determined by flow cytometry for: RFP+ or GFP+ transduced cells and EpCAM-APC+ primary tumor cells.

Gating strategy

Primary EpCAM+ tumor cells: morphology (FSC/SSC) -> single cells (FSC-A/FSC-H)-> live cells (PI-) -> EpCAM-APC+ tumor cells
shRNA or Cre transduced human or mouse pancreatic cancer cells: morphology (FSC/SSC) -> singe cells (FSC-A/FSC-H)-> live cells (PI-) -> GFP+ or RFP+ vector+ cells. Primary bulk tumor cells: morphology (FSC/SSC) -> single cells (FSC-A/FSC-H)-> live cells (PI-) -> CD45- -> CD31- -> PDGFRa- -> EpCAM+ or EpCAM-
Boundaries between "positive" and "negative" populations were defined using a fluorescence minus one sample.

- [x] Tick this box to confirm that a figure exemplifying the gating strategy is provided in the Supplementary Information.
